# Psychological resilience and burnout in nursing interns versus registered nurses: a systematic review and meta-analysis

**DOI:** 10.3389/fpsyt.2026.1920134

**Published:** 2026-10-14

**Authors:** Xiaomei Chen, Yiyun Zhang, Fangfang Zhang, Xiaodi Zhu

**Affiliations:** Second Ward, Department of Traditional Chinese Medicine, The First Affiliated Hospital of Anhui Medical University, Hefei, Anhui, China

**Keywords:** burnout, education-to-practice transition, emotional exhaustion, meta-analysis, nurses, nursing interns, nursing students, psychological resilience

## Abstract

**Background:**

Burnout is a common, serious occupational mental-health problem in nursing, and psychological resilience is a modifiable factor that may be associated with lower burnout. Whether this association holds equally across the education-to-practice transition, from interns to registered nurses, is unclear, and the most comparable prior meta-analysis excluded nursing students and the Chinese-language literature.

**Objective:**

We aimed to pool the resilience–burnout association in nurses and nursing interns. Two coprimary outcomes were prespecified: the resilience–emotional-exhaustion (EE) correlation, and a concept-matched intern-versus-registered-nurse comparison on total burnout. Secondary outcomes were depersonalization and personal accomplishment.

**Methods:**

Nine databases, five English-language and four Chinese-language, were searched from inception to 2026 with no language filter applied inside any database; eligibility was limited to reports published in English or Chinese. Eligible studies were cross-sectional and reported a correlation between a validated resilience measure and burnout in nurses or nursing interns. Two reviewers independently screened, extracted data, and appraised quality (AHRQ 11-item). Correlations were pooled via Fisher’s *z* under random-effects models (REML; metafor), with subgroup, sensitivity, and small-study analyses.

**Results:**

Seventy-eight occupational-burnout studies were analyzed (29,292 participants); five academic-burnout studies were out of scope. Resilience was inversely associated with emotional exhaustion (*r* = − 0.36, 95% CI = − 0.42 to − 0.31; *k* = 49; *I*^2^ = 95%), total burnout (− 0.44), and depersonalization (− 0.30), and positively associated with personal accomplishment (+ 0.43). On the concept-matched comparison (total burnout), registered nurses showed a numerically stronger association than interns (*r* = − 0.46 vs. − 0.28; *Q*_between *p* = 0.025); with only five intern studies and instability in sensitivity analyses, this difference is preliminary. Estimates were stable across leave-one-out, model-choice, and PET-PEESE analyses, and no weaker in the nine studies scoring AHRQ ≥ 8 (*r* = − 0.43, − 0.59 to − 0.24); no funnel-plot asymmetry was detected (Egger’s *p* = 0.49).

**Conclusions:**

Psychological resilience is a moderate, consistent protective correlate of nurse burnout, but a wide prediction interval reflecting high heterogeneity means its practical benefit likely varies across settings. Resilience-building should complement, not replace, organizational measures. Whether the correlate is stronger in registered nurses than in interns remains preliminary.

**Systematic Review Registration:**

https://www.crd.york.ac.uk/PROSPERO/view/CRD420261427008, identifier CRD420261427008.

## 1 Introduction

Burnout is a work-related psychological syndrome that arises from chronic occupational stress. It is classically defined along three dimensions: emotional exhaustion (the depletion of emotional and physical resources), depersonalization or cynicism (a detached, callous response to recipients of care), and reduced personal accomplishment (a sense of ineffectiveness at work) (1). Nurses work at the frontline of emotionally demanding, high-acuity care, and burnout is common in this group. It harms not only their own mental and physical health but also the safety and quality of the care they provide.

Nursing burnout is a global problem. A meta-analysis of 94 studies across more than 30 countries estimated a pooled burnout prevalence of about 30% over the past decade, and this prevalence rose during the coronavirus disease 2019 (COVID-19) pandemic (2). Its consequences are also well documented. A recent synthesis of 85 studies and more than 288,000 nurses linked nurse burnout to poorer patient safety, lower patient satisfaction, and reduced quality of care (3). Identifying modifiable factors that can protect nurses against burnout has therefore become a research and workforce-policy priority.

Psychological resilience is the capacity to adapt positively in the face of adversity, stress, or trauma. It has emerged as one such protective factor, and because it can be developed through training, it is a plausible target for intervention across the nursing career. That career, however, is not uniform. Nursing interns (students on clinical internship or practicum) face their first clinical exposure and the well-described transition or reality shock (4–6), whereas registered nurses face the chronic, cumulative stressors of established practice. Whether resilience protects equally at these two stages, across the education-to-practice transition, bears directly on when and for whom resilience-building education should be prioritized.

Prior reviews have begun to quantify the resilience–burnout link, but a key gap remains. The most directly comparable meta-analysis (7) pooled the association in nurses (overall *r* ≈ − 0.41) from 29 studies. That review, however, searched only four English-language databases through early 2022, was restricted to English, and, most importantly, excluded nursing students and interns. It therefore could not test whether the protective association differs across the education-to-practice transition. No previous synthesis has included clinically exposed nursing interns alongside registered nurses or formally tested a between-group difference in the resilience–burnout association. The large body of Chinese-language evidence has also been largely missing from international syntheses, which raises concerns about completeness and language bias.

To address these gaps, we conducted a systematic review and meta-analysis with three aims. First, we pooled the correlation between psychological resilience and burnout, with emotional exhaustion as the primary burnout dimension, across both nursing interns and registered nurses. Second, we carried out what is, to our knowledge, the first concept-matched between-group comparison (a test for subgroup differences) of this association across the education-to-practice transition. Third, we broadened the evidence base by searching nine Chinese and English databases through 2026, applying no language filter at the search stage while restricting eligibility to reports published in English or Chinese. Our directional hypothesis, grounded in Conservation of Resources theory and the transition-shock literature, was that resilience would be inversely associated with burnout in both groups but that the association would be stronger in registered nurses than in nursing interns: registered nurses have consolidated the professional identity, coping repertoire, and resource “caravans” through which trait resilience is thought to exert its protective effect, whereas interns, who are still undergoing transition shock and acquiring professional resources, may not yet translate resilience as fully into protection.

## 2 Methods

### 2.1 Registration and reporting

This systematic review and meta-analysis was prospectively registered in the International Prospective Register of Systematic Reviews (PROSPERO; registration number CRD420261427008, registered on 17 June 2026) and is reported in accordance with the Preferred Reporting Items for Systematic Reviews and Meta-Analyses (PRISMA 2020) statement (8). The literature searches had commenced before the protocol was formally registered, and registration was completed before any data analysis was undertaken; the final search-update date was 19 June 2026, and the manuscript was submitted on 26 June 2026. The protocol was followed with a single documented amendment, reported transparently: because occupational-burnout intern studies almost never reported emotional exhaustion, the prespecified between-group test (initially specified on emotional exhaustion) was conducted on total burnout, the construct with adequate data in both groups (**Section 3.4**); all other analyses followed the registered protocol. As the study synthesized previously published, de-identified aggregate data, ethical approval was not required.

### 2.2 Eligibility criteria (PECOS)

Eligibility was framed using the PECOS principle.

Participants (P) comprised two clinically exposed groups: (A) nursing interns (students on clinical internship/practicum) and (B) registered nurses in clinical practice; preclinical students not yet on placement were excluded, as work-related burnout does not apply, as were mixed samples of other health professions (e.g., physicians, nursing assistants, midwives) that could not be disaggregated to nurses. The exposure (E) was psychological resilience, measured with any validated resilience instrument (e.g., Connor–Davidson Resilience Scale [CD-RISC 25/10/2] (9), Brief Resilience Scale, Wagnild and Young Resilience Scale [RS-14/RS-25], Resilience Scale for Adults, and validated Chinese adaptations); the resilience instrument was examined as a prespecified moderator and sensitivity factor. The comparison (C) was nursing interns (group A) versus registered nurses (group B); for effect-size pooling, no comparator is required, and group served as the primary moderator. The outcome (O) was occupational (work-related) burnout, with studies measuring academic or study-related burnout (e.g., Maslach Burnout Inventory (MBI)–Student Survey in nonclinical students) outside the review’s scope and excluded from the main analysis (reported separately). Within occupational burnout, a prespecified outcome hierarchy avoided ambiguity: the primary outcome was the resilience–emotional exhaustion (EE) correlation, EE being the core, most consistently reported burnout dimension and the most comparable construct across instruments and across both groups, and the anchor of the overall synthesis. Secondary outcomes were the resilience correlations with (i) total or global burnout (the overall burnout score as operationalized by each study’s instrument, e.g., MBI summed score, Chinese Maslach Burnout Inventory (CMBI) total, Copenhagen Burnout Inventory [CBI] total, Professional Quality of Life Scale (ProQOL) burnout subscale burnout subscale, Oldenburg [OLBI] total), (ii) depersonalization/cynicism (DP), and (iii) personal accomplishment (PA). Each outcome was pooled in a separate meta-analysis, and the negatively keyed dimensions (EE, DP, total) and the positively keyed PA were never combined into one estimate. As instruments (MBI–Human Services Survey (MBI-HSS)/MBI–General Survey (MBI-GS)/MBI–Student Survey (MBI-SS), CMBI, CBI, ProQOL, OLBI) operationalize burnout differently (most acutely for “total burnout”, which is not identical across instruments), the burnout instrument was recorded for every study and examined as a prespecified moderator and as a sensitivity restriction (MBI-only). Eligible study designs (S) were cross-sectional or observational correlational studies reporting a zero-order Pearson (or Spearman) correlation coefficient (*r*) between resilience and burnout, or sufficient statistics to derive it. For clarity, a nursing intern was defined as a preregistration nursing student undergoing supervised clinical placement; preclinical students without placement and newly graduated, licensed nurses in independent practice were not classified as interns (the latter were included as registered nurses, group B). The occupational-versus-academic classification for every intern and student sample is given in **Supplementary Table S4**.

Exclusion criteria: (1) reviews, qualitative studies, intervention/experimental studies (RCT/quasi-experiment), case reports, conference abstracts, editorials, and letters; (2) studies measuring a conceptually distinct construct (moral resilience only) in place of general psychological resilience (analyzed separately, not pooled) or compassion fatigue (ProQOL burnout subscale handled as a burnout measure where reported); (3) studies not reporting an extractable correlation coefficient and sample size (regression/SEM/ORs without a derivable zero-order *r*); (4) duplicate cohorts, including the same cohort published in different languages: suspected duplicates were adjudicated by matching author overlap, sample size, city/hospital, data-collection period, instruments, and the pattern of reported coefficients against the full texts, and only the most complete report was retained; (5) reports not published in English or Chinese, the two languages in which the review team could appraise a full text and extract data without translation; (6) predatory or non-peer-reviewed sources, defined operationally by source type rather than prestige as (a) popular-science or consumer-health periodicals, (b) digest or abstracting compilations, (c) open-access outlets meeting objective indicators of predatory practice, and (d) clearly off-field (e.g., secondary-education) periodicals—all legitimate peer-reviewed journals, including lower-tier ones, were retained and addressed only through the quality subgroup and sensitivity analyses; (7) reports for which the full text could not be obtained despite interlibrary-loan and author-contact attempts.

### 2.3 Information sources and search strategy

Nine databases were searched from inception to 19 June 2026: PubMed (*n* = 1,236), Embase (*n* = 1,039), Web of Science (*n* = 2,087), Cumulative Index to Nursing and Allied Health Literature (CINAHL) (*n* = 1,428), and the Cochrane Library (*n* = 147) (English); and China National Knowledge Infrastructure (CNKI, *n* = 127), Wanfang (*n* = 364), VIP (*n* = 130) and SinoMed/CBM (*n* = 328) (Chinese), yielding 6,886 records before deduplication. The search combined three concept blocks (psychological resilience × nurses × burnout) using database-specific controlled vocabulary (MeSH, Emtree, CINAHL headings) and free-text terms (full strategies for all nine databases in **Supplementary Appendix A**). Study design was not restricted in the search and was applied at full-text screening to maximize sensitivity. Reference lists of included studies and relevant reviews were hand-searched (backward snowballing), and forward citation tracking was performed. No language filter was applied inside any of the nine databases, four of which index the Chinese-language literature, and Chinese-language reports were sought and retrieved on the same terms as English-language ones. Language entered the review only as an eligibility criterion, because the team could appraise a full text and extract data reliably only in English and Chinese. No report was excluded on language grounds at the full-text stage (**Figure 1**).

**Figure 1:**
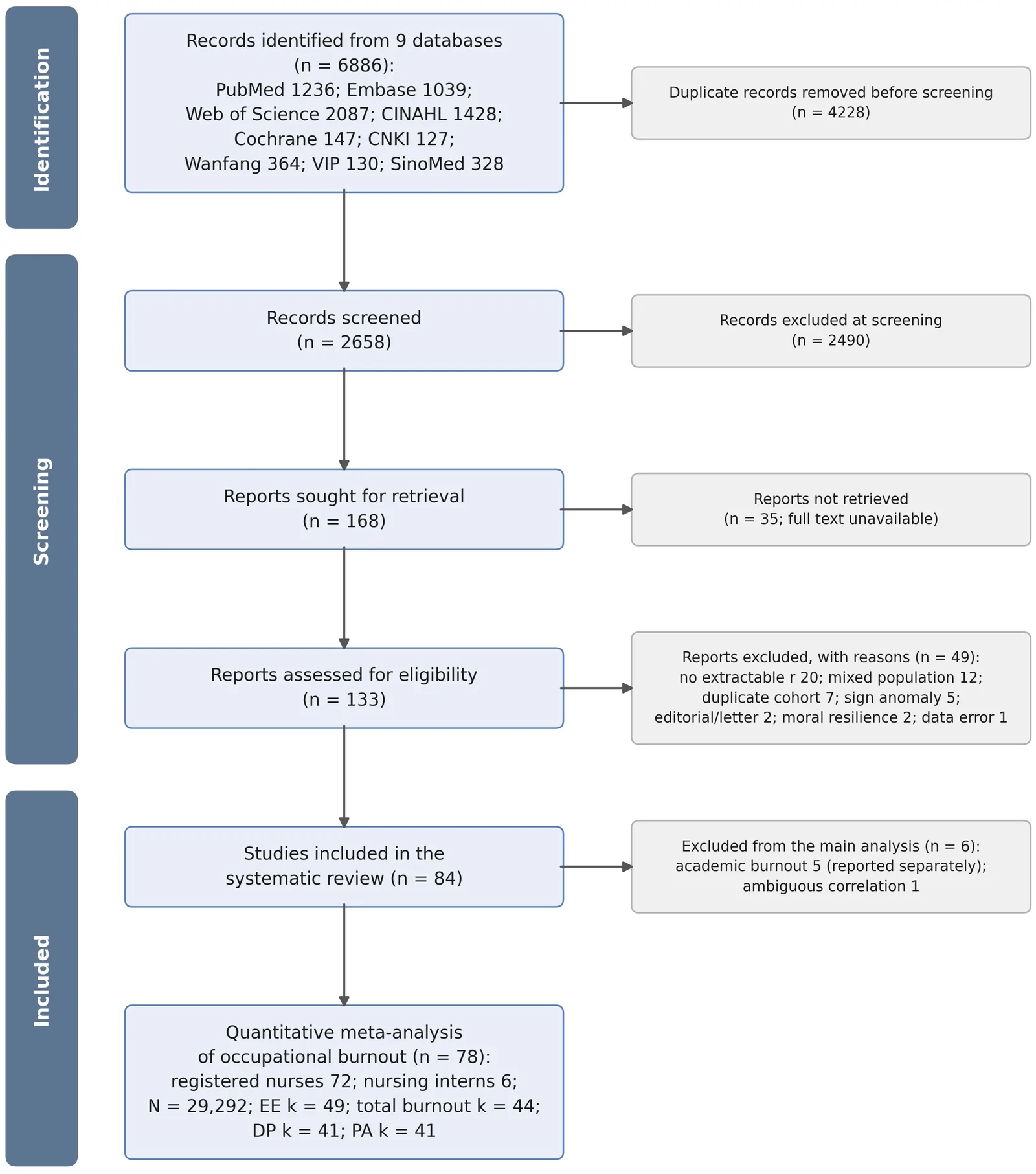
PRISMA 2020 flow diagram of study identification and selection.

### 2.4 Study selection and data extraction

Records were imported into reference-management software, deduplicated, and screened independently by two reviewers against the eligibility criteria, first by title/abstract and then by full text; disagreements were resolved by discussion or a third reviewer. The selection process is summarized in a PRISMA 2020 flow diagram (**Figure 1**). As part of deduplication, we specifically screened for cross-language twins (the same cohort reported in Chinese and in English) using the matching procedure above; where the same cohort had been reported more than once, the more complete report was retained, and same-author clusters that were in fact distinct samples (for example, Guo 2018/2019 and the Ríos-Risquez/García-Izquierdo studies, which differed in sample size, coefficients, and DOIs) were kept separately.

Two reviewers independently extracted the following information into a piloted spreadsheet: first author, publication year, country/region, setting/specialty, population (nurse vs. nursing student), sampling, sample size, resilience instrument, burnout instrument, and the correlation coefficient(s) between resilience and burnout (total and by subscale). Where a study reported only regression coefficients, mediation/SEM path coefficients, or odds ratios without a zero-order correlation, the bivariate *r* was sought from correlation tables; studies reporting neither *r* nor convertible statistics were excluded. Discrepancies were reconciled against the source article. A minority of studies reported a Spearman rather than a Pearson coefficient; these were pooled with the Pearson coefficients and, in a prespecified sensitivity analysis, excluded, which did not materially change the pooled estimate. Where a zero-order Pearson *r* was not reported directly, it was derived using standard conversions (for example, from a standardized regression coefficient), and the number of studies requiring any such derivation was recorded in the extraction sheet.

### 2.5 Risk of bias (quality) assessment

Methodological quality of the included cross-sectional studies was independently appraised by two reviewers using the Agency for Healthcare Research and Quality (AHRQ) 11-item cross-sectional checklist; per the registered protocol, total scores classified studies as high (8–11), moderate (6, 7), or low (≤ 5) quality. The JBI Critical Appraisal Checklist for analytical cross-sectional studies was used as a secondary appraisal. Disagreements were resolved by consensus. The overall certainty of the pooled evidence for each outcome was rated using the GRADE approach (10). Throughout the manuscript, “high-quality” refers to this AHRQ threshold (total score ≥ 8); where studies are instead described by source type (indexed, core-journal, or thesis), the term “core-source” is used to keep the two senses distinct. Quality entered the analysis in two ways: the three AHRQ grades were compared in a test for subgroup differences, and the primary outcome was repooled with the analysis restricted to studies scoring 8 or more and, separately, to studies scoring 6 or more. The restriction by source type is reported alongside these and is not a quality restriction.

### 2.6 Statistical analysis

Analyses were performed in R (version ≥ 4.3) using the metafor package (11). The effect measure was the Pearson correlation coefficient (*r*) between psychological resilience and burnout. As *r* is bounded and its variance depends on its magnitude, each *r* was transformed to Fisher’s *z* (the standard approach for meta-analysis of correlations) (12), pooled on the *z* scale, and back-transformed to *r* for interpretation. Given anticipated clinical and methodological diversity, a random-effects model (restricted maximum likelihood [REML]) was used *a priori*. Separate pooled estimates were computed for resilience with (i) total burnout, (ii) emotional exhaustion, (iii) depersonalization/cynicism, and (iv) personal accomplishment; the personal-accomplishment dimension (a positively keyed construct) was analyzed separately and not combined with the exhaustion/depersonalization dimensions.

Heterogeneity was quantified with the *Q* test and *I*^2^ statistic (interpreted per the registered bands: 0%–25% low, 26%–50% moderate, 51%–100% high) (13), and a 95% prediction interval was reported. The review’s central inferential step was a formal test for subgroup differences (*Q_between*) comparing the resilience–burnout association between nursing interns (group A) and registered nurses (group B). As occupational-burnout intern studies rarely reported the emotional-exhaustion dimension (*k* = 1), the between-group test was conducted on total burnout (a documented amendment to the registered protocol, which had specified emotional exhaustion). As the main analysis was restricted to occupational burnout, this comparison is construct-matched by design (both groups measured work-related burnout). Additional prespecified subgroup analyses examined: region (China vs. other), resilience instrument, burnout instrument, and survey period (pre- vs. post-COVID-19). Where ≥10 studies were available, meta-regression explored continuous moderators (publication year, sample size; mean age and % female were too inconsistently reported to model). Sensitivity analyses comprised leave-one-out analysis and comparison of fixed- versus random-effects estimates. Publication bias was assessed (when ≥ 10 studies) by funnel-plot inspection and Egger’s regression test, with trim-and-fill applied if asymmetry was detected. Forest plots, leave-one-out plots, and funnel plots were generated in metafor. Statistical significance was set at *p* < 0.05 (*p* < 0.10 for heterogeneity and bias tests). These registered *I*^2^ bands differ from the conventional Cochrane thresholds; we retain them as preregistered and, to avoid over-reliance on *I*^2^, foreground *τ*² and the 95% prediction interval for every pooled estimate. Small-study effects were additionally examined with PET-PEESE.

## 3 Results

### 3.1 Study selection and characteristics

Of the records screened, 84 cross-sectional reports met the eligibility criteria for the systematic review. Eighty-three of these reported an extractable zero-order correlation between psychological resilience and burnout and are characterized in **Table 1** (14–96); the remaining study was excluded from the quantitative synthesis because it reported an internally inconsistent, unverifiable total-burnout coefficient. As this review concerns occupational (work-related) burnout, five of these 83 studies were conducted in nursing students and measured academic (study-related) burnout; being out of scope, they were excluded from the main analysis and are reported separately in **Section 3.6**. The main analysis therefore comprised 78 studies of occupational burnout with 29,292 participants (sample sizes: 25–1,627), whose characteristics are summarized in **Table 1**. Studies were published between 2009 and 2026 and conducted in China (*n* = 26) and other countries or regions (*n* = 52). Most samples were registered nurses (group B, *k* = 72); clinically exposed nursing interns (group A) contributed six occupational-burnout studies. A separate moral-resilience subgroup (*k* = 2) was analyzed apart from the main psychological-resilience pool. Resilience was most often measured with the CD-RISC family (*k* = 51; CD-RISC-25/CD-RISC-10/CD-RISC-2), followed by the Brief Resilience Scale (*k* = 9), the Wagnild and Young Resilience Scale (*k* = 5), and other validated instruments (*k* = 13, including two hardiness and two work-resilience scales). Burnout was assessed predominantly with the Maslach Burnout Inventory family (MBI-GS, MBI-HSS, MBI-other, MBI-SS; *k* ≈ 49); the remaining studies used CBI, ProQOL, OLBI/OBI, or single-dimension exhaustion measures. Of the analyzable studies, 60 were published in 2020 or later (during or after the COVID-19 pandemic) and 18 before. Among the 78 studies, methodological quality (AHRQ 11-item) was high in 13, moderate in 38, and low in 27 (mean ≈ 6.2/11); the full item-level appraisal is given in **Supplementary Table S1**. The high proportion of low ratings reflects the structural limits of cross-sectional self-report designs on this checklist, where items on assessor blinding, follow-up, and random sampling are rarely met. Thirteen of the 78 studies scored 8 or more, and nine of those 13 reported an emotional-exhaustion coefficient. We therefore report the pooled estimate stratified by study quality in the main analysis (below) as well as in the sensitivity analyses (**Section 3.6**). The GRADE certainty of the pooled evidence was low to very low for all outcomes, downgraded for serious inconsistency and risk of bias (Summary of findings; **Table 2**).

**Table 1:** Characteristics and AHRQ methodological quality of the included studies (*k* = 83; main occupational-burnout analysis, *k* = 78).

Study	Region	Group^a^	*N*	Resilience	Burnout	*r*_total	*r*_EE	*r*_DP	*r*_PA	AHRQ	Quality
Gao et al. (14)	China	RN	857	CD-RISC	CMBI	− 0.613				7	Moderate
Chai et al. (15)	China	RN	233	CD-RISC	MBI-other	− 0.661	− 0.743	− 0.672	0.677	8	High
Yun et al. (16)	China	RN	845	Work	MBI-other	− 0.525				4	Low
Gao et al. (17)	China	RN	717	Other	MBI-GS	− 0.636	− 0.528	− 0.636	0.549	7	Moderate
Huang et al. (18)	China	RN	1,579	CD-RISC	MBI-HSS		− 0.361	− 0.237	0.556	6	Moderate
Yan et al. (19)	China	RN	1,627	BRS	EE-only		− 0.484			5	Low
Lyu (20)	China	RN	265	CD-RISC	ProQOL	− 0.203				7	Moderate
Yang (21)	China	RN	300	CD-RISC	MBI-GS		− 0.132	− 0.158	0.486	8	High
Guo (22)	China	RN	299	CD-RISC	CMBI		− 0.31	− 0.359	0.324	7	Moderate
Sun (23)	China	RN	165	CD-RISC	MBI-HSS		− 0.054	− 0.212	0.626	8	High
Sun (24)	China	RN	352	CD-RISC	MBI-other		− 0.741	− 0.698	0.703	8	High
Ke et al. (25)	China	RN	1,574	CD-RISC	MBI-GS	− 0.571				7	Moderate
Chen et al. (26)	China	RN	527	CD-RISC	EE-only		− 0.582			7	Moderate
Cui et al. (27)	China	RN	730	Other	MBI-other	− 0.49	− 0.34	− 0.39	0.37	5	Low
Zou et al. (28)	China	RN	375	CD-RISC	MBI-GS	− 0.449	− 0.222	− 0.25	0.448	6	Moderate
He and Zhang (29)	China	RN	703	Hardiness	MBI-other		− 0.182	− 0.397	0.351	6	Moderate
Ou (30)	China	RN	225	CD-RISC	MBI-GS	− 0.15	− 0.11	− 0.24	0.04	8	High
Huang (31)	China	RN	394	CD-RISC	MBI-GS	− 0.567	− 0.287	− 0.402	0.549	7	Moderate
Zhou (32)	China	RN	203	CD-RISC	MBI-GS	− 0.47	− 0.14	− 0.12	0.66	7	Moderate
Li (33)	China	RN	192	CD-RISC	MBI-GS	− 0.495	− 0.405	− 0.39	0.379	5	Low
Al-Harrasi et al. (34)	Other	RN	173	BRS	MBI-HSS		− 0.26	− 0.176	0.218	7	Moderate
Georges et al. (35)	Other	RN	179	CD-RISC	MBI-HSS		− 0.17	− 0.007	0.263	5	Low
Yildirim et al. (36)	Other	RN	201	BRS	Other	− 0.496				5	Low
Guo et al. (37)	China	RN	1,061	CD-RISC	MBI-GS		− 0.271	− 0.311	0.317	6	Moderate
Lang et al. (38)	Other	RN	266	CD-RISC	MBI-GS	− 0.126				8	High
Rushton et al. (39)	Other	RN	114	CD-RISC	MBI-HSS		− 0.31	− 0.23	0.59	4	Low
Luo et al. (40)	China	RN	458	RS	MBI-HSS	− 0.51	− 0.43	− 0.33	0.39	9	High
Yang et al. (41)	China	RN	536	CD-RISC	MBI-other		− 0.1	− 0.12	0.42	7	Moderate
Lee et al. (42)	Other	RN	605	CD-RISC	CBI	− 0.323				5	Low
Rivas et al. (43)	Other	RN	101	CD-RISC	MBI-other		− 0.271		0.288	4	Low
Zhang et al.^b^ (44)	China	Intern	635	CD-RISC	Other	− 0.57				7	Moderate
Jiménez-Fernández et al. (45)	Other	RN	375	CD-RISC	MBI-other		− 0.332	− 0.2	0.484	5	Low
Abdulmohdi (46)	Other	RN	111	CD-RISC	OLBI/OBI	− 0.227	− 0.269	− 0.145		6	Moderate
Ochs et al. (47)	Other	RN	25	CD-RISC	MBI-student/educ		− 0.631	− 0.619	0.491	3	Low
Merino-Godoy et al. (48)	Other	Intern	393	CD-RISC	EE-only	− 0.189				3	Low
Abram and Jacobowitz (49)	Other	RN	51	RS	ProQOL	− 0.39				6	Moderate
Ríos-Risquez et al.^b^ (50)	Other	Intern	218	CD-RISC	MBI-SS		− 0.33	− 0.16	0.44	7	Moderate
Zhang et al. (51)	China	RN	319	CD-RISC	MBI-other		− 0.365	− 0.301	0.287	8	High
Alkrenawi et al. (52)	Other	Intern	105	CD-RISC	ProQOL	− 0.27				7	Moderate
Kolutek et al. (53)	Other	RN	945	CD-RISC	CBI	− 0.4				8	High
LaPlante et al. (54)	Other	RN	218	CD-RISC	Other	− 0.314				7	Moderate
Zhang et al. (55)	China	RN	180	CD-RISC	MBI-GS	− 0.387	− 0.283	− 0.4	0.388	5	Low
Arrogante and Aparicio-Zaldívar (56)	Other	RN	133	CD-RISC	MBI-HSS		− 0.26	− 0.29	0.37	7	Moderate
Guo et al. (57)	China	RN	197	CD-RISC	MBI-GS	− 0.382	− 0.258	− 0.254	0.428	7	Moderate
Alkubati et al. (58)	Other	RN	306	BRS	MBI-other		− 0.48	− 0.34	0.16	8	High
Min (59)	Other	RN	155	Other	MBI-other	− 0.58				5	Low
Ogińska-Bulik and Michalska (60)	Other	RN	72	Other	OLBI/OBI	− 0.59	− 0.5			7	Moderate
García-Izquierdo et al. (61)	Other	RN	537	CD-RISC	MBI-GS		− 0.11	− 0.22	0.22	4	Low
Jiménez-García et al. (62)	Other	RN	256	CD-RISC	MBI-HSS		− 0.373	− 0.169	0.392	5	Low
Gensimore et al. (63)	Other	RN	501	CD-RISC	MBI-HSS		− 0.272	− 0.265	0.367	6	Moderate
Ríos-Risquez et al.^b^ (64)	Other	Intern	113	CD-RISC	MBI-SS		− 0.51	− 0.2	0.35	7	Moderate
Cha et al. (65)	Other	RN	271	CD-RISC	MBI-GS	− 0.589				6	Moderate
Jeong and Shin (66)	Other	RN	147	Other	MBI-other	− 0.54				6	Moderate
Vieira et al. (67)	Other	RN	153	Work	MBI-other		− 0.545	− 0.419	0.68	5	Low
Sikaras et al. (68)	Other	RN	380	BRS	CBI	− 0.457				6	Moderate
Jose et al. (69)	Other	RN	120	CD-RISC	MBI-HSS		− 0.252	− 0.069	0.313	5	Low
Zahednezhad et al. (70)	Other	RN	202	CD-RISC	MBI-other	− 0.5	− 0.46	− 0.26	0.37	6	Moderate
Souza-Costa et al. (71)	Other	RN	239	CD-RISC	MBI-other		− 0.428	− 0.291	0.52	5	Low
Rees et al. (72)	Other	Intern	415	CD-RISC	ProQOL	− 0.486				7	Moderate
Lee et al. (73)	Other	RN	146	CD-RISC	MBI-other	− 0.48				7	Moderate
Jamebozorgi et al. (74)	Other	RN	364	CD-RISC	MBI-HSS		− 0.442		0.351	5	Low
Kutluturkan et al. (75)	Other	RN	140	RS	MBI-other		− 0.191	− 0.161	0.35	6	Moderate
Kchaou et al. (76)	Other	RN	106	BRS	ProQOL	− 0.71				7	Moderate
Baugh et al. (77)	Other	RN	27	BRS	MBI-GS		− 0.179	− 0.272	0.503	4	Low
Hwang and Lee (78)	Other	RN	131	CD-RISC	MBI-other	− 0.51				8	High
Shalom et al. (79)	Other	RN	465	CD-RISC	CBI	− 0.282				6	Moderate
Miyazaki et al. (80)	Other	RN	351	Other	Other		− 0.278	− 0.295		6	Moderate
Altharman et al. (81)	Other	Intern	564	CD-RISC	MBI-other		− 0.11	− 0.12	0.43	5	Low
Suazo Galdames et al. (82)	Other	RN	1,013	RS	MBI-HSS		− 0.121	− 0.114	0.439	5	Low
Lee et al. (83)	Other	Intern	140	Other	MBI-GS	− 0.36				6	Moderate
Alia et al.^b^ (84)	Other	Intern	244	Other	Other	− 0.296				6	Moderate
Buljubašić et al. (85)	Other	RN	125	BRS	Other	− 0.258				5	Low
Zanjani et al. (86)	Other	RN	108	CD-RISC	Other	− 0.38				5	Low
Mohammadi et al. (87)	Other	RN	420	Other	Other		− 0.7			7	Moderate
Aghaei and Asadi (88)	Other	RN	712	Other	MBI-other	− 0.25				9	High
Rísquez et al.^b^ (89)	Other	Intern	218	CD-RISC	MBI-GS		− 0.248	− 0.063	0.521	6	Moderate
Ghoneam et al. (90)	Other	Intern	780	CD-RISC	CBI	− 0.097				7	Moderate
Rubaca and Munir (91)	Other	RN	426	BRS	EE-only		− 0.618			7	Moderate
Ata et al. (92)	Other	RN	79	RS	ProQOL	− 0.424				6	Moderate
Kim et al. (93)	Other	RN	144	CD-RISC	EE-only		− 0.511			8	High
Ertem et al. (94)	Other	RN	55	CD-RISC	Other	− 0.542	− 0.707	− 0.351	0.567	4	Low
Alonazi et al. (95)	Other	RN	177	CD-RISC	ProQOL	− 0.21				5	Low
Ko et al. (96)	Other	RN	157	Hardiness	ProQOL	− 0.68				7	Moderate

N, sample size; r_total, correlation of resilience with total burnout; r_EE, correlation of resilience with emotional exhaustion; r_DP, correlation of resilience with depersonalization; r_PA, correlation of resilience with personal accomplishment; blank cells = not reported; AHRQ, Agency for Healthcare Research and Quality 11-item score (quality rated high [≥ 8], moderate [6–7], or low [≤ 5]); CD-RISC, Connor–Davidson Resilience Scale; MBI, Maslach Burnout Inventory; CBI, Copenhagen Burnout Inventory.

^a^ Group A = nursing interns; group B = registered nurses.

^b^ Academic-burnout study (nursing students, classified within group A), reported separately in Section 3.6 and not included in the 78-study occupational-burnout analysis. The full version with journal and region is provided in **Supplementary Table S3**.

**Table 2:** GRADE summary of findings.

Outcome (resilience ↔ …)	*k*	Participants	Pooled *r* (95% CI)	*I* ^2^	Certainty (GRADE)	Reasons for rating
Emotional exhaustion (primary)	49	18,473	− 0.36 (− 0.42, − 0.31)	95%	Low–very low	Start low (observational); − 1 serious inconsistency (*I*^2^ = 95%, PI − 0.67 to + 0.05); − 1 serious risk of bias (mean AHRQ ≈ 6.2/11; 27/78 low). No imprecision (large *k*, narrow CI); no statistical evidence of funnel asymmetry (Egger’s *p* = 0.49). Not upgraded (moderate, not large, effect)
Total burnout	44	15,163	− 0.44 (− 0.48, − 0.39)	92%	Low–very low	As above (serious inconsistency + risk of bias)
Depersonalization/cynicism	41	14,792	− 0.30 (− 0.35, − 0.24)	91%	Low–very low	As above
Personal accomplishment (sign-harmonized)	41	14,795	+0.43 (+0.38, +0.48)	91%	Low–very low	As above

GRADE, Grading of Recommendations Assessment, Development and Evaluation; CI, confidence interval; PI, prediction interval; EE, emotional exhaustion; DP, depersonalization; PA, personal accomplishment. Certainty of evidence was downgraded for risk of bias, inconsistency (high I^2^).

### 3.2 Primary outcome: resilience and emotional exhaustion

Across 49 occupational-burnout studies, psychological resilience was inversely associated with emotional exhaustion: pooled *r* = − 0.36 (95% confidence interval [CI] = − 0.42 to − 0.31; more precisely = − 0.364, − 0.418 to − 0.306; *p* < 0.001; **Figure 2**). Heterogeneity was high (*I*^2^ = 95%), and the 95% prediction interval (− 0.67 to + 0.05) indicates that, although the average association is moderate, the true association in an individual setting could range from strong to near-null. Restricted to registered nurses (group B, *k* = 48), the estimate was essentially unchanged (*r* = − 0.37, 95% CI = − 0.42 to − 0.31; *I*^2^ = 94%). Restricting to the studies rated high quality on the AHRQ checklist, meaning a total score of 8 or more, left nine emotional-exhaustion studies, in which the association was, if anything, stronger: *r* = − 0.43, 95% CI = − 0.59 to − 0.24, *I*^2^ = 97%. Dropping only the low-scoring studies (AHRQ ≥ 6) gave *r* = − 0.37, 95% CI = − 0.45 to − 0.29, *k* = 30. AHRQ grade was not a significant moderator, *Q* = 1.37, *df* = 2, *p* = 0.51, so the pooled result does not depend on the lower-quality studies.

**Figure 2:**
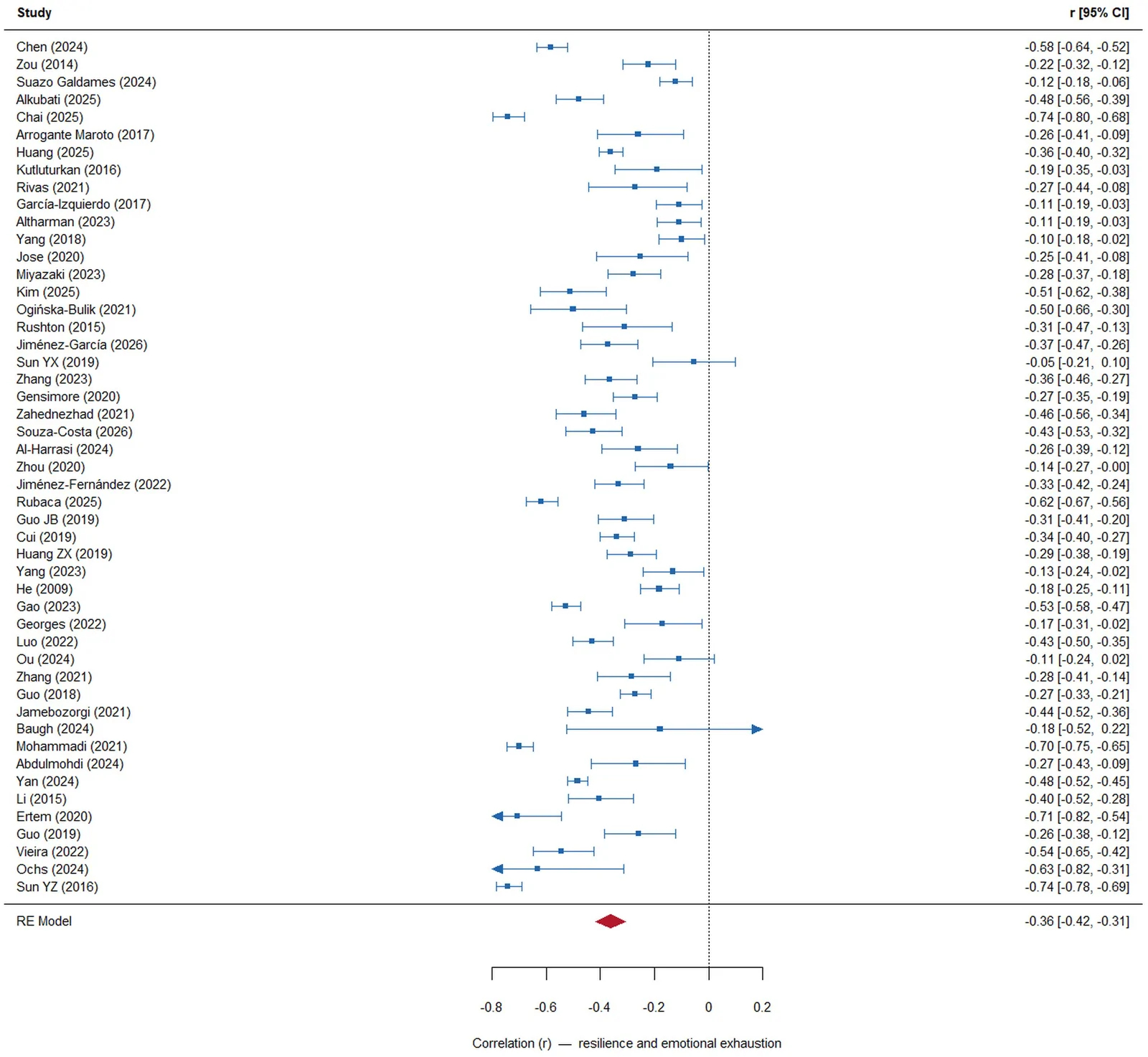
Forest plot of the association between psychological resilience and emotional exhaustion (random-effects model; *k* = 49).

### 3.3 Secondary burnout outcomes

Resilience was inversely associated with total burnout (*k* = 44; *r* = − 0.44, 95% CI = − 0.48 to − 0.39; *I*^2^ = 92%; PI = − 0.69 to − 0.10; **Supplementary Figure S1**) and with depersonalization/cynicism (DP; *k* = 41; *r* = − 0.30, 95% CI = − 0.35 to − 0.24; *I*^2^ = 91%), and positively associated with personal accomplishment (PA; *k* = 41; *r* = + 0.43, 95% CI = + 0.38 to + 0.48; *I*^2^ = 91%; signs harmonized so that higher PA = greater accomplishment). Here, *N* is the total number of unique participants across all included studies, each study counted once; the participant counts underlying each outcome (EE = 18,473; total burnout = 15,163; DP = 14,792; PA = 14,795) are subsets of that total, because most studies reported some but not all dimensions, and are not intended to sum to *N* (**Table 2**).

Thus, resilience was negatively associated with the two “negative” burnout dimensions (EE, DP) and positively associated with personal accomplishment, consistent with a protective pattern across all components.

### 3.4 Key comparison: nursing interns versus registered nurses (group moderator)

As the main analysis was restricted to occupational burnout, the intern–nurse comparison is concept-matched by design: both groups measured work-related burnout (**Supplementary Table S2**). For total burnout, the outcome with adequate data in both groups, registered nurses showed a numerically stronger association (*r* = − 0.46, *k* = 39, 95% CI = − 0.50 to − 0.41) than nursing interns (*r* = − 0.28, *k* = 5, 95% CI = − 0.42 to − 0.13); *Q*_between = 5.04, *df* = 1, *p* = 0.025. This difference should be interpreted as preliminary and hypothesis-generating, for three reasons. First, the intern arm rests on only five studies, and an intern-arm leave-one-out analysis showed the pooled intern estimate moving between *r* = − 0.21 and *r* = − 0.33 depending on which single study was removed. Second, the burnout instrument was itself a significant moderator (see **Section 3.5**), so the career-stage contrast is partly confounded with instrument mix; in a group-plus-instrument meta-regression, the group difference persisted but was attenuated (*β* = − 0.19, *p* = 0.040). Third, the single intern study using a single-dimension emotional-exhaustion-only scale was treated, per the prespecified rule, as contributing an overall-burnout indicator to this analysis; reassigning it instead to the EE dimension reduced the intern arm to four studies and rendered the difference nonsignificant (*p* = 0.083). The EE-based comparison could not be made directly, because only one occupational-burnout intern study reported EE as a subscale of a multidimensional instrument; the between-group test was therefore amended in advance to total burnout (**Section 2.6**). The pattern is directionally consistent with a strengthening of the protective association across the education-to-practice transition, but is statistically fragile and needs confirmation in larger intern samples.

### 3.5 Moderators and subgroup analyses (EE)

All subgroup and moderator results are summarized in **Table 3**.

**Table 3:** Subgroup and moderator analyses (resilience and emotional exhaustion).

Moderator/subgroup	*k*	Pooled *r* (95% CI)	Test of difference
Group: total burnout (construct-matched, primary comparison)			
Nursing interns (A)	5	− 0.283 (− 0.421, − 0.133)	*Q_*between = 5.04, *df* = 1, *p* = 0.025
Registered nurses (B)	39	− 0.457 (− 0.504, − 0.408)	
Resilience instrument (EE)			
CD-RISC	34	− 0.343 (− 0.409, − 0.272)	*Q*_mod, *p* = 0.31
Brief Resilience Scale	5	− 0.446 (− 0.574, − 0.297)	
Wagnild and Young RS	3	− 0.254 (− 0.437, − 0.050)	
Other	5	− 0.484 (− 0.626, − 0.311)	
Region (EE)			
China	22	− 0.353 (− 0.440, − 0.260)	*Q*_mod, *p* = 0.74
Other countries	27	− 0.372 (− 0.441, − 0.298)	
Burnout instrument (EE)			
Single-dimension EE scale	4	− 0.551 (− 0.613, − 0.482)	*Q*_mod, *p* = 0.003; *R*^2^ ≈ 21%
MBI-GS	12	− 0.254 (− 0.334, − 0.171)	
MBI-HSS	12	− 0.284 (− 0.352, − 0.212)	
MBI-other	14	− 0.400 (− 0.513, − 0.273)	
Other	7	− 0.496 (− 0.637, − 0.324)	
Survey period (EE)			
Post-COVID-19 (≥ 2020)	34	− 0.399 (− 0.462, − 0.332)	*Q*_mod, *p* = 0.056; *R*^2^ ≈ 6%
Pre-COVID-19 (< 2020)	15	− 0.283 (− 0.378, − 0.181)	
Study quality (AHRQ grade, EE)			
High (AHRQ ≥ 8)	9	− 0.431 (− 0.592, − 0.236)	*Q*_mod, *p* = 0.51
Moderate (AHRQ 6–7)	21	− 0.350 (− 0.425, − 0.269)	
Low (AHRQ ≤ 5)	19	− 0.341 (− 0.414, − 0.264)	
Continuous meta-regression (EE)			By year, *p* ≈ 0.07; by *N*, *p* = 0.80 (both nonsignificant)

The intern-versus-registered-nurse comparison on EE was not estimable because only one occupational-burnout intern study reported EE. All estimates are for the resilience–emotional-exhaustion association, except the group comparison, which is for total burnout (the construct-matched outcome with adequate data in both groups). In the resilience-instrument subgroup, two studies using construct-divergent scales (hardiness and work-specific resilience) are not shown and are examined in the construct-purification sensitivity analysis (Section 3.6); the remaining subgroup counts sum to the 49-study analysis total.

*k*, number of studies; *r*, pooled Pearson correlation (random-effects, REML); *95% CI*, 95% confidence interval; *I^2^*, heterogeneity statistic; *Q_between*, between-subgroup heterogeneity test; *R^2^*, proportion of between-study variance explained.

The resilience instrument did not significantly moderate the EE association (*Q*_mod, *p* = 0.31; CD-RISC *r* = − 0.34, *k* = 34; Brief Resilience Scale (BRS) *r* = − 0.45, *k* = 5; Wagnild and Young RS *r* = − 0.25, *k* = 3; other *r* = − 0.48, *k* = 5), and neither did region (China vs. other: *p* = 0.74; China − 0.35, *k* = 22; other − 0.37, *k* = 27). The burnout instrument was a significant moderator (*Q*_mod, *p* = 0.003, *R*^2^ ≈ 21%): single-dimension EE measures showed the strongest association (*r* = − 0.55, *k* = 4), whereas MBI-GS (*r* = − 0.25, *k* = 12) and MBI-HSS (*r* = − 0.28, *k* = 12) were weaker, making the instrument a notable source of heterogeneity. The COVID-19 period was no longer a significant moderator after removal of the duplicate cohort (*p* = 0.056), with postpandemic studies tending to show a stronger association (*r* = − 0.40, *k* = 34) than prepandemic studies (*r* = − 0.28, *k* = 15); because absence of statistical significance is not evidence of equivalence, this should be read as inconclusive rather than as showing no period effect. On prespecified continuous meta-regression, the resilience–EE association was not significantly related to publication year (*p* = 0.07) or sample size (*p* = 0.80), providing no evidence of small-study effects on the continuous scale. Subgroups containing only three or four studies (e.g., the Wagnild and Young and single-dimension EE strata) carry wide confidence intervals and should be interpreted with corresponding caution.

### 3.6 Sensitivity analyses and robustness

The primary EE estimate was stable across all sensitivity analyses (**Table 4**).

**Table 4:** Sensitivity analyses (resilience and emotional exhaustion).

Analysis	*k*	Pooled *r* (95% CI)	*I* ^2^	Note
Main model (random-effects, REML)	49	− 0.364 (− 0.418, − 0.306)	95%	Primary estimate; 95% PI − 0.67 to + 0.05
Fixed-effect model	49	− 0.358	–	Concordant with random-effects
Leave-one-out (range of pooled *r*)	49	− 0.352 to − 0.369	–	No single study materially alters the estimate
CD-RISC instruments only	34	− 0.343 (− 0.409, − 0.272)	94%	Conclusion held
High methodological quality only (AHRQ ≥ 8)	9	− 0.431 (− 0.592, − 0.236)	97%	Association no weaker than in the full pool; wide CI, small *k*
Low-quality studies excluded (AHRQ ≥ 6)	30	− 0.374 (− 0.449, − 0.295)	96%	Conclusion held
Low-quality studies only (AHRQ ≤ 5)	19	− 0.341 (− 0.414, − 0.264)	91%	Conclusion held
Core-source reports only (indexed or core journals and theses)	45	− 0.345 (− 0.400, − 0.287)	93%	Restriction by source type, not by methodological quality
CD-RISC × AHRQ ≥ 8 (strictest subset)	7	− 0.423 (− 0.628, − 0.162)	97%	Conclusion held
Construct-pure (excluding hardiness/work-resilience scales)	–	Essentially unchanged	–	Not driven by construct-divergent scales
Trim-and-fill (publication bias)	49	− 0.364	–	0 studies imputed; Egger’s *z* = − 0.69, *p* = 0.49

All estimates are random-effects (REML) models; the primary EE estimate is shown for comparison in each row. Two distinct restrictions are reported, and they are not interchangeable. The AHRQ rows restrict the analysis based on methodological quality scored with the 11-item checklist, whereas the core-source row restricts the analysis based on publication source type, meaning indexed journals, core journals, and theses, and carries no quality score. Of the 78 occupational-burnout studies, 13 scored 8 or more on AHRQ, and nine of those reported an emotional-exhaustion coefficient, so that row is the least precise in the table.

EE, emotional exhaustion; CD-RISC, Connor–Davidson Resilience Scale; r, pooled correlation; 95% CI, 95% confidence interval; k, number of studies.

Leave-one-out analysis left the pooled r within a narrow range (− 0.352 to − 0.369; **Figure 3**). Fixed- and random-effects estimates were nearly identical (*r* = − 0.358 vs. − 0.364). Small-study-adjusted estimates were consistent with the primary result: PET gave an adjusted *r* = − 0.32 (95% CI = − 0.45 to − 0.18), and PEESE gave *r* = − 0.35 (− 0.42 to − 0.27), both clearly negative. All conclusions held when the pool was restricted to CD-RISC instruments (*r* = − 0.34, *k* = 34), studies scoring AHRQ ≥ 8 (*r* = − 0.43, *k* = 9), studies scoring AHRQ ≥ 6 (*r* = − 0.37, *k* = 30), and core-source reports, meaning indexed or core journals and theses (*r* = − 0.34, *k* = 45). The last of these restricts the analysis based on the type of source rather than on methodological quality, and the two criteria are reported separately in **Table 4**. Construct purification, which excluded studies using hardiness or work-specific resilience scales and retained only general psychological-resilience instruments, also left estimates essentially unchanged, indicating that the pooled associations were not driven by construct-divergent scales. As a precautionary sensitivity analysis for total burnout, excluding one study (Gao 2023) whose total-burnout coefficient was identical to its depersonalization coefficient and could not be reverified against the source left the pooled estimate essentially unchanged (*r* = − 0.43, *k* = 43) and the intern-versus-registered-nurse difference significant (*Q*_between *p* = 0.028).

**Figure 3:**
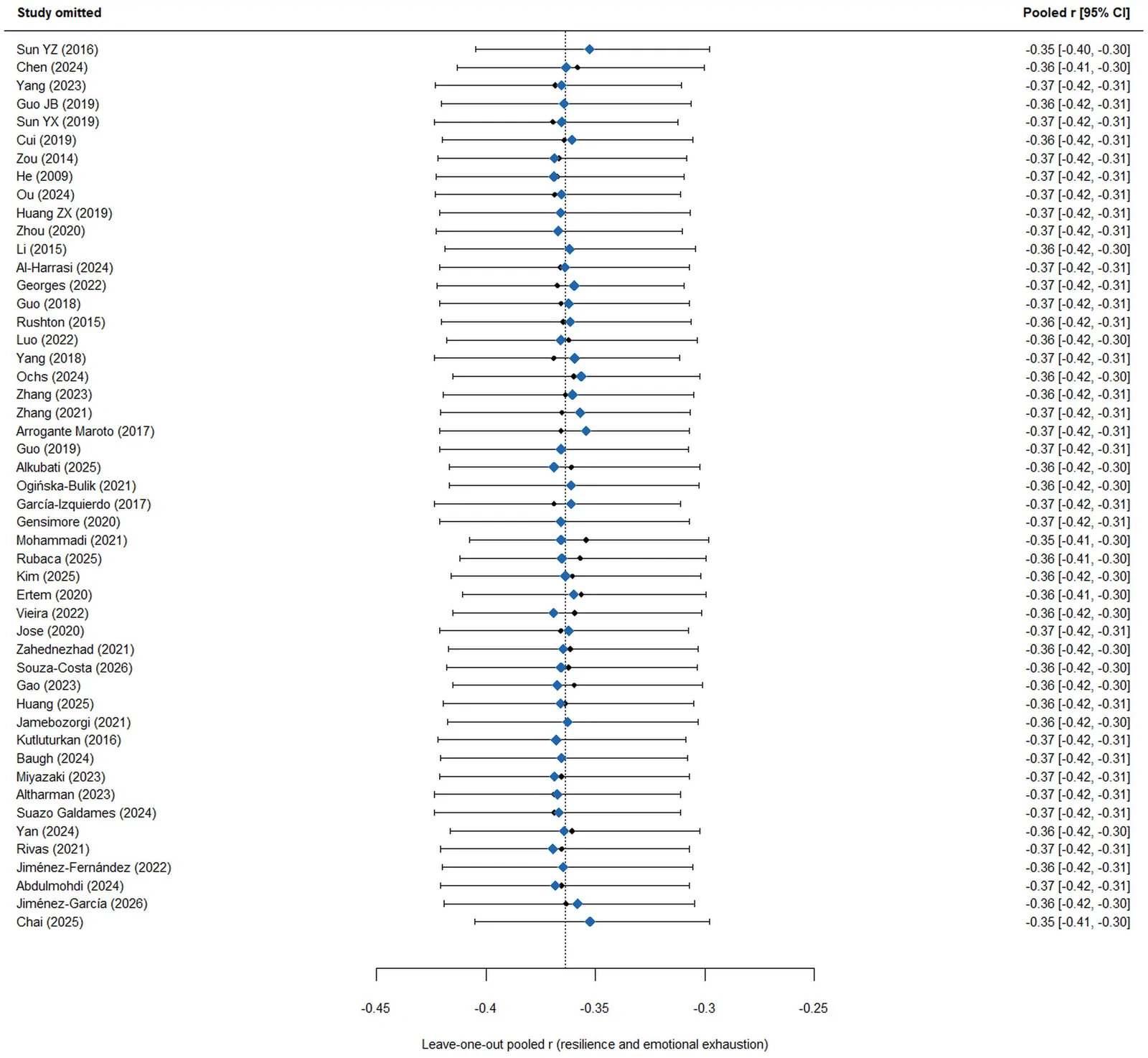
Leave-one-out sensitivity analysis for the resilience–emotional-exhaustion association.

#### 3.6.1 Academic-burnout subgroup (out of main scope, reported separately)

The five excluded nursing-student studies measuring academic burnout showed an association of similar magnitude (resilience–academic-exhaustion *r* = − 0.36, *k* = 3), but academic and occupational burnout are distinct constructs and were not pooled.

### 3.7 Publication bias

For the primary EE outcome, no statistical evidence of funnel-plot asymmetry was detected (Egger’s regression test *z* = − 0.69, *p* = 0.49) and trim-and-fill imputed no missing studies (adjusted *r* unchanged at − 0.36; **Figure 4**). These tests have low power and limited validity under extreme heterogeneity, however, so the absence of detected asymmetry does not establish the absence of publication or retrieval bias; as a further check, small-study-adjusted PET-PEESE estimates (Section 3.6) remained clearly negative.

**Figure 4:**
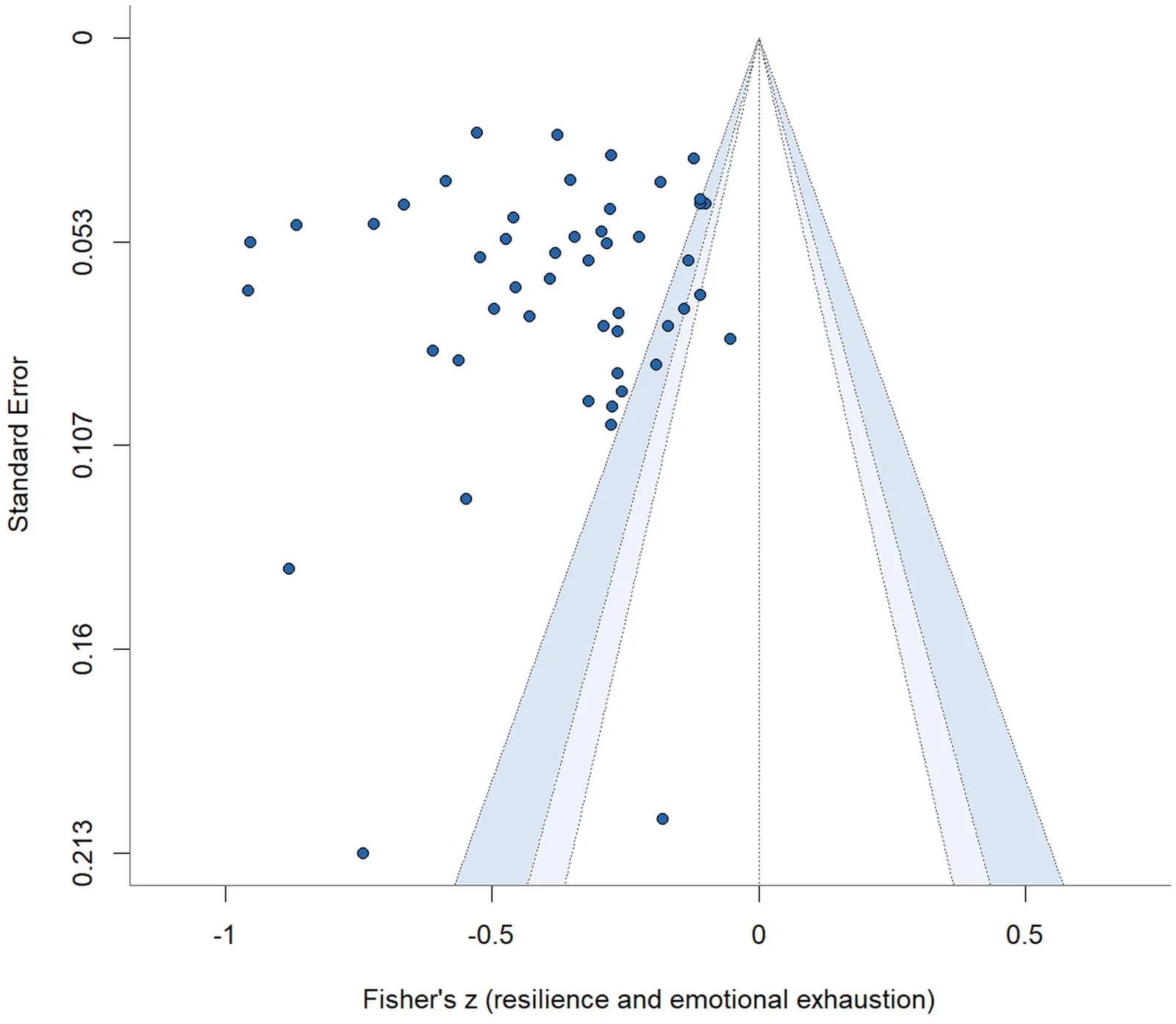
Funnel plot for the resilience–emotional-exhaustion association.

## 4 Discussion

This meta-analysis of 78 cross-sectional studies of occupational burnout (29,292 participants) found a moderate and consistent inverse association between psychological resilience and burnout in nurses and clinically exposed nursing interns. Resilience was inversely associated with emotional exhaustion (pooled *r* = − 0.36), total burnout (− 0.44), and depersonalization (− 0.30), and positively associated with personal accomplishment (+ 0.43). The direction and size of these associations agree closely with the most comparable prior synthesis (overall *r* = − 0.41; exhaustion − 0.27; depersonalization − 0.23). As our evidence base is roughly three times larger, multilingual, and current to 2026, this agreement corroborates and updates the earlier estimate. Our included literature (78 studies) is well within the norm for correlational nursing syntheses and comparable in scope to other recent high-impact reviews (97, 98).

Resilience showed its most consistently reported inverse association with emotional exhaustion (*r* = − 0.36, 95% CI = − 0.42 to − 0.31; *k* = 49), and the estimate was almost identical when restricted to registered nurses (− 0.37). This exceeds the pooled exhaustion correlation of the comparator meta-analysis (− 0.27), plausibly because of our broader inclusion of Chinese cohorts and post-pandemic studies, in which the association tended to be stronger. Several pathways are consistent with, though not demonstrated by, this cross-sectional association: resilient individuals tend to report more adaptive stress responses, and resilience is associated with cognitive reappraisal and active coping that may limit emotional depletion, as well as with help-seeking and the mobilization of social support. As the design is cross-sectional, these mechanisms are hypotheses rather than established pathways. Practically, resilience-strengthening programs (for example, mindfulness, cognitive reappraisal, and peer support) are a plausible component of exhaustion-reduction efforts and could be built into both prelicensure curricula and continuing professional development, while recognizing that their benefit is likely to vary across settings.

The protective pattern was coherent across all burnout components. Resilience was negatively associated with the two negative dimensions, exhaustion and depersonalization, and positively associated with personal accomplishment (+ 0.43). The weaker association with depersonalization (− 0.30) mirrors prior work. It may be that depersonalization is driven more by organizational and relational factors, such as workplace incivility and staffing, than by individual adaptive capacity, whereas accomplishment is closely tied to the self-efficacy that resilience builds.

Our main prespecified question was whether the strength of the association differs across the education-to-practice transition. As the analysis was confined to occupational burnout, the comparison is concept-matched by design: both groups measured work-related burnout, which avoids the academic-versus-occupational confound. On total burnout, registered nurses showed a numerically stronger association than nursing interns (*r* = − 0.46 vs. − 0.28; *Q*_between *p* = 0.025). This is consistent with the hypothesis, grounded in Conservation of Resources theory and the transition-shock literature, that resilience operates within a more consolidated professional identity and resource structure in registered nurses, whereas for interns, who are still accumulating professional resources and face the acute novelty of first clinical exposure, it may be only one of several competing influences. The signal is fragile, however: it rests on only five intern studies, the intern estimate is unstable on leave-one-out, it is attenuated after adjusting for the burnout instrument, and it becomes nonsignificant if a single study is reclassified (**Section 3.4**). We therefore treat it as a preliminary, hypothesis-generating result and frame our contribution here as methodological—to our knowledge, the first concept-matched between-group comparison of this association across career stages—rather than as an established effect. In practical terms, the pattern is compatible with stage-tailored approaches (transition-shock and expectation-management support for interns, sustained reinforcement for practicing nurses), but confirmation will require adequately powered intern samples.

Heterogeneity was high (*I*^2^ ≈ 95%), as is typical for correlational meta-analyses, and we examined several moderators of the exhaustion association. The burnout instrument was a significant source of heterogeneity (*Q*_mod *p* = 0.003, *R*^2^ ≈ 21%): single-dimension exhaustion scales gave the strongest associations (− 0.55), whereas MBI-GS (− 0.25) and MBI-HSS (− 0.28) gave weaker ones. This points to the need for measurement harmonization. By contrast, the resilience instrument and region were not significant moderators, and the COVID-19 period was no longer significant after removal of the duplicate cohort (*p* = 0.056); because absence of evidence is not evidence of absence, these null moderator results (which may reflect limited power) should not be read as demonstrating equivalence. More importantly, the wide prediction interval for emotional exhaustion (− 0.67 to + 0.05) means that in some settings the true association could be close to zero: the pooled value is a benchmark, not a parameter that applies uniformly, and resilience interventions cannot be assumed to be equally effective across all contexts and populations. Policymakers, managers, and clinicians should therefore treat resilience-building as one locally evaluated element of a broader strategy rather than as a guaranteed lever.

The review has several strengths. It was prospectively registered with PROSPERO. The nine-database bilingual search, run without a language filter and covering the Chinese as well as the English literature, assembled one of the largest and most current evidence bases for this association to date, recovering a substantial Chinese literature absent from earlier syntheses. Emotional exhaustion was treated, *a priori*, as the primary and most comparable outcome. The synthesis provides what is, to our knowledge, the first formal between-group comparison across the education-to-practice transition, supported by a broad set of robustness checks. The primary estimate was stable under leave-one-out and under fixed- versus random-effects models, and it held in the analyses that address quality directly. Restricting to CD-RISC-only studies gave − 0.34, restricting to the nine studies scoring AHRQ ≥ 8 gave − 0.43, and dropping the low-scoring studies, AHRQ ≥ 6, gave − 0.37; a further restriction to core-source reports, a criterion of source type rather than of quality, gave − 0.34. The high-quality subset is small, and its confidence interval correspondingly wide, but the association in it is no weaker than in the full pool. Egger’s test (*p* = 0.49) and trim-and-fill (no studies imputed) showed no evidence of publication or small-study bias.

Several limitations qualify these findings. First, all included studies were cross-sectional, which precludes causal inference; reverse causation (lower burnout permitting higher self-rated resilience) and common-method variance (both constructs self-reported) cannot be excluded. Second, heterogeneity was very high (*I*^2^ ≈ 91%–95%) and only partly explained (burnout instrument *R*^2^ ≈ 21%). The pooled point estimate should therefore be read as a benchmark rather than a population parameter, alongside the prediction interval, which for emotional exhaustion (− 0.67 to + 0.05) indicates that the true association could be near-null in some settings. Accordingly, GRADE certainty is low to very low: observational designs, downgraded for serious inconsistency and risk of bias (**Table 2**). Third, the intern arm was small (*k* = 5 for total burnout) and unstable, so the between-group difference is preliminary and is not robust to reasonable reclassification of a single study; occupational-burnout intern studies also rarely reported emotional exhaustion, so the EE-based comparison could not be performed. Fourth, eligibility was limited to reports published in English or Chinese, so evidence published in other languages could not be appraised, and some Chinese non-core-source studies could not be retrieved in full text and were excluded; Egger’s test detects only within-pool small-study asymmetry and is blind to systematically unretrieved literature, so residual retrieval and language bias cannot be excluded, although the main analysis already incorporates substantial Chinese full-text evidence, and small-study-adjusted PET-PEESE estimates remained clearly negative. Fifth, the personal-accomplishment dimension required transparent sign harmonization across studies that reported reduced accomplishment.

As resilience is modifiable through training (workplace resilience-training trials and meta-analyses report small-to-moderate reductions in distress) (99, 100), these findings translate into concrete, stage-specific recommendations.

In the prelicensure curriculum, rather than a stand-alone lecture, resilience competencies (cognitive reappraisal, help-seeking, realistic expectation-setting) should be embedded in clinical-placement preparation and in structured postplacement debriefs and reflective practice, where they match the demands interns actually face. Across the education-to-practice transition, resilience and reality-shock inoculation should be integrated into transition-to-practice or nurse-residency and preceptorship programs for newly graduated nurses, grounded in transition-shock theory (4, 5) and the novice-to-expert trajectory (6). For management and retention, because depersonalization is driven more by organizational than individual factors, individual resilience-building must be paired with system-level action on workload, staffing, civility, and psychological safety; under Conservation of Resources and Job Demands–Resources theory (101, 102), resilience is a personal resource that may complement, but cannot substitute for, reductions in job demands.

**Ethical caveat**. Framing burnout prevention mainly as individual resilience risks placing responsibility for a structural problem on practitioners. Our recommendations therefore treat resilience interventions as a complement to, not a substitute for, organizational reform. Future research should prioritize longitudinal and intervention designs that can establish temporal precedence, report emotional-exhaustion effect sizes for nursing interns specifically, use concept-matched (occupational) burnout measures across career stages, and adopt standardized instruments to reduce measurement-driven heterogeneity.

## 5 Conclusion

In this meta-analysis of 78 studies of occupational burnout (29,292 nurses and nursing interns), psychological resilience was a moderate and consistent protective correlate of burnout (emotional exhaustion *r* = − 0.36; total burnout *r* = − 0.44). The estimates were stable across extensive sensitivity, small-study-adjusted, and subgroup analyses, but they carry low-to-very-low GRADE certainty and a wide prediction interval owing to high heterogeneity, so the practical benefit of resilience is likely to vary across settings. On the concept-matched between-group comparison (total burnout), registered nurses showed a numerically stronger association than nursing interns (*Q*_between *p* = 0.025); this is a preliminary, hypothesis-generating signal, based on only five intern studies and not robust to sensitivity analyses, that the value of resilience may strengthen across the education-to-practice transition, and it needs confirmation in adequately powered intern samples. Resilience-building may be a reasonable but not universally sufficient component of a broader strategy—tentatively transition support for interns and sustained reinforcement for practicing nurses—and should complement, not replace, organizational measures to reduce burnout.

## Data Availability

The original contributions presented in the study are included in the article/**Supplementary Material**. Further inquiries can be directed to the corresponding author.

## Supplementary material

SUPPLEMENTARY APPENDIX A Search strategies and results across the nine databases.

SUPPLEMENTARY TABLE S1 AHRQ item-level quality appraisal of included studies.

SUPPLEMENTARY TABLE S2 Group × burnout-construct cross-tabulation.

SUPPLEMENTARY TABLE S3 Characteristics and AHRQ methodological quality of all included studies (full version, with journal and region; k = 83).

SUPPLEMENTARY TABLE S4 Occupational-versus-academic burnout classification decision table for intern and student samples.

SUPPLEMENTARY FIGURE S1 Forest plot of the association between psychological resilience and total burnout (random-effects model; k = 44).

## References

[1] Maslach C Jackson SE . The measurement of experienced burnout. J Organ Behav. (1981) 2:99–113. doi: 10.1002/job.403002020542587375

[2] Ge MW Hu FH Jia YJ Tang W Zhang WQ Chen HL . Global prevalence of nursing burnout syndrome and temporal trends for the last 10 years: a meta-analysis of 94 studies covering over 30 countries. J Clin Nurs. (2023) 32:5836–54. doi: 10.1111/jocn.16708 37194138

[3] Li LZ Shanafelt T . Nurse burnout and patient safety, satisfaction, and quality of care: a systematic review and meta-analysis. JAMA Netw Open. (2024) 7:e2443059. doi: 10.1001/jamanetworkopen.2024.43059 39499515 PMC11539016

[4] Duchscher JEB . Transition shock: the initial stage of role adaptation for newly graduated registered nurses. J Adv Nurs. (2009) 65:1103–13. doi: 10.1111/j.1365-2648.2008.04898.x 19183235

[5] Kramer M . Reality shock: why nurses leave nursing. St. Louis, MO: C.V. Mosby. (1974).

[6] Benner P . From novice to expert: excellence and power in clinical nursing practice. Menlo Park, CA: Addison-Wesley.. (1984).

[7] Castillo-González A . Relation and effect of resilience on burnout in nurses: a literature review and meta-analysis. Int Nurs Rev. (2024) 71:160–7. doi: 10.1111/inr.12838 37000679

[8] Page MJ McKenzie JE Bossuyt PM Boutron I Hoffmann TC Mulrow CD . The PRISMA 2020 statement: an updated guideline for reporting systematic reviews. BMJ. (2021) 372:n71. doi: 10.1136/bmj.n71 33782057 PMC8005924

[9] Connor KM Davidson JRT . Development of a new resilience scale: the Connor-Davidson Resilience Scale (CD-RISC). Depress Anxiety. (2003) 18:76–82. doi: 10.1002/da.10113 12964174

[10] Guyatt G Oxman AD Akl EA Kunz R Vist G Brozek J . GRADE guidelines: 1. Introduction-GRADE evidence profiles and summary of findings tables. J Clin Epidemiol. (2011) 64:383–94. doi: 10.1016/j.jclinepi.2010.04.026 21195583

[11] Viechtbauer W . Conducting meta-analyses in R with the metafor package. J Stat Softw. (2010) 36:1–48. doi: 10.18637/jss.v036.i03

[12] Borenstein M Hedges LV Higgins JPT Rothstein HR . Introduction to Meta-Analysis. Chichester, UK: John Wiley & Sons. (2009).

[13] Higgins JPT Thompson SG Deeks JJ Altman DG . Measuring inconsistency in meta-analyses. BMJ. (2003) 327:557–60. doi: 10.1136/bmj.327.7414.557 12958120 PMC192859

[14] Gao Y Ren H Meng G Ying X Jin P Chen Y . The chain mediating role of resilience and change fatigue between intolerance of uncertainty and job burnout in nurses. Sci Rep. (2025) 15:22469. doi: 10.1038/s41598-025-06803-2 40595102 PMC12216394

[15] Chai F Hu W Xu D Ji Y Wang Y Xu C . The relationship between moral distress and occupational burnout among ICU nurses: social support and psychological resilience as mediating variables. Front Public Health. (2025) 13:1743774. doi: 10.3389/fpubh.2025.1743774 41602075 PMC12833362

[16] Yun Z Zhou P Zhang B . High-performance work systems, thriving at work, and job burnout among nurses in Chinese public hospitals: the role of resilience at work. Healthc (Basel). (2022) 10:1935. doi: 10.3390/healthcare10101935 36292382 PMC9601832

[17] Gao F Chui PL Che CC Mao XR . Nurses’ burnout, resilience, and its associated factors in Sichuan, China, during the peak of coronavirus disease 2019 infection. Florence Nightingale J Nurs. (2023) 31:152–9. doi: 10.5152/fnjn.2023.23023 37847013 PMC10724793

[18] Huang Y Wang Z Li Y Zhao Z Wang W Cai C . Anxiety and burnout in infectious disease nurses: the role of perceived stress and resilience. BMC Nurs. (2025) 24:3. doi: 10.1186/s12912-024-02649-7 39754082 PMC11697665

[19] Yan W Chen X Xiao D Wang H Du X Li L . Patient mistreatment, social sharing of negative events and emotional exhaustion among chinese nurses: the combined moderating effect of organizational support and trait resilience. BMC Nurs. (2024) 23:260. doi: 10.1186/s12912-024-01924-x 38649852 PMC11036568

[20] Lyu MH . Analysis of the correlation between compassion fatigue and emotional exhaustion, psychological resilience, and the nursing work environment among emergency department nurses. [Master's thesis]. Kunming, China: Kunming Medical University; (2023). doi: 10.27202/d.cnki.gkmyc.2023.001355

[21] Yang ZY . A study on the relationship among social support, psychological resilience, and job burnout in elderly-care nursing staff. [Master's thesis]. Ganzhou, China: Gannan Medical University; (2023). doi: 10.27959/d.cnki.ggnyx.2023.000118

[22] Guo JB . Transition shock and job burnout among new nurses: the moderating role of psychological resilience and social support. [Master's thesis]. Kaifeng, China: Henan University; (2019).

[23] Sun YX . A study on the association of occupational burnout with psychological resilience and positive emotion among operating room nurses. [Master's thesis]. Jinan, China: Shandong University; (2019). [in Chinese]

[24] Sun YZ . The mediating effect of psychological resilience between work stress and job burnout among clinical nurses. [Master's thesis]. Hefei, China: Anhui Medical University; (2016). [in Chinese]

[25] Ke L Xu LL Li Y Li LT Jia J . Chain mediating effect of perceived social support and resilience between occupational burnout and depression among clinical nurses. J Nurs Sci. (2024) 39:56–60. doi: 10.3870/j.issn.1001-4152.2024.15.056

[26] Chen TL Sun SJ Zhou Q Chen YF . The moderating effect of psychological resilience between emotional labor and emotional exhaustion among nurses. Mil Nurs. (2024) 41:47–50. doi: 10.3969/j.issn.2097-1826.2024.05.01242277785

[27] Cui S Zhang LJ Yang HZ Li XM Zhao Y . The mediating role of psychological resilience between perceived stress and occupational burnout among clinical nurses. Chin Nurs Res. (2019) 33:1244–7. doi: 10.12102/j.issn.1009-6493.2019.07.038

[28] Zou GY Shen XY Tian XH Liu CQ Li P . The mediating role of psychological resilience between occupational burnout and positive coping among clinical nurses. J Nurses Train. (2014) 29:1830–3.

[29] He LL Zhang P . A correlational study on job burnout and hardy personality among nurses. J Nurs Sci. (2009) 24:52–4. doi: 10.3870/hlxzz.2009.20.052

[30] Ou N . A correlation and mediation study of occupational burnout, moral distress, and psychological resilience among ICU nurses. [Master's thesis]. Suzhou, China: Soochow University; (2024). [in Chinese]

[31] Huang ZX . The impact of workplace violence on nurses' occupational burnout: the mediating role of psychological resilience. [Master's thesis]. Guangzhou, China: Jinan University; (2019). [in Chinese]

[32] Zhou YZ . The effect of adult attachment styles on occupational burnout among intensive care unit nurses: psychological resilience as a moderating variable. [Master's thesis]. Hangzhou, China: Zhejiang University; (2020). [in Chinese]

[33] Li HF Liu CQ Li P . The relationship between psychological resilience and job burnout in psychiatric nurses: the mediating role of positive emotions. Chin J Nurs. (2015) 50:1083–6. doi: 10.3761/j.issn.0254-1769.2015.09.013

[34] Al-Harrasi S Sabei SA Omari OA Abrawi UA . Nurses’ job burnout and resilience in neonatal intensive care units. J Perinat Neonatal Nurs. (2024) 38:201–11. doi: 10.1097/jpn.0000000000000817 38758275

[35] Georges MT Roberts LR Johnston Taylor E Nick JM Dehom S . Burnout, self-efficacy, and resilience in Haitian nurses: a cross-sectional study. J Holist Nurs. (2022) 40:310–25. doi: 10.1177/08980101211065600 34951321

[36] Yildirim D Şenyuva E Kaya E . Turkish nurses’ psychological resilience and burnout levels during the COVID-19 pandemic: a correlational study. Int J Nurs Pr. (2024) 30:e13239. doi: 10.1111/ijn.13239 38308441

[37] Guo YF Luo YH Lam L Cross W Plummer V Zhang JP . Burnout and its association with resilience in nurses: a cross-sectional study. J Clin Nurs. (2018) 27:441–9. doi: 10.1111/jocn.13952 28677270

[38] Lang M Jones L Harvey C Munday J . Workplace bullying, burnout and resilience amongst perioperative nurses in Australia: a descriptive correlational study. J Nurs Manag. (2021) 30:1502–13. doi: 10.1111/jonm.13437 34350645

[39] Rushton CH Batcheller J Schroeder K Donohue P . Burnout and resilience among nurses practicing in high-intensity settings. Am J Crit Care. (2015) 24:412–20. doi: 10.4037/ajcc2015291 26330434

[40] Luo D Song Y Cai X Li R Bai Y Chen B . Nurse managers’ burnout and organizational support: the serial mediating role of leadership and resilience. J Nurs Manag. (2022) 30:4251–61. doi: 10.1111/jonm.13852 36205051

[41] Yang G Liu J Liu L Wu X Ding S Xie J . Burnout and resilience among transplant nurses in 22 hospitals in China. Transpl Proc. (2018) 50:2905–10. doi: 10.1016/j.transproceed.2018.04.033 30348454

[42] Lee WT Hsu HC Lee HF Efendi F Ramoo V Hung HM . Empowerment, resilience, and burnout among nurses with recovered COVID-19: a multicountry study. J Nurs Manag. (2025) 2025:6861497. doi: 10.1155/jonm/6861497 41424993 PMC12714158

[43] Rivas N López M Castro MJ Luis-Vian S Fernández-Castro M Cao M . Analysis of burnout syndrome and resilience in nurses throughout the COVID-19 pandemic: a cross-sectional study. Int J Env Res Public Health. (2021) 18:10470. doi: 10.3390/ijerph181910470 34639769 PMC8508575

[44] Zhang L Zhang Q Li S Li Y Wu G Chen Y . The mediating role of psychological resilience in Chinese nursing students’ professional identity and learning burnout. J Korean Acad Nurs. (2024) 54:509. doi: 10.4040/jkan.24044 39663616

[45] Jiménez-Fernández R Corral-Liria I Trevissón-Redondo B Lopez-Lopez D . Burnout, resilience and psychological flexibility in frontline nurses during the acute phase of the COVID-19 pandemic (2020) in Madrid, Spain. J Nurs Manag. (2022) 30:2549–56. doi: 10.1111/jonm.13778 36042534 PMC9539113

[46] Abdulmohdi N . The relationships between nurses’ resilience, burnout, perceived organisational support and social support during the second wave of the COVID-19 pandemic: a quantitative cross-sectional survey. Nurs Open. (2024) 11:e2036. doi: 10.1002/nop2.2036 38268251 PMC10697858

[47] Ochs J Mitsakis C Ochberg R . A correlational study measuring burnout and resilience among nurse educators. Nurs Educ Perspect. (2024) 46:106–8. doi: 10.1097/01.nep.0000000000001288 38819222

[48] Merino-Godoy MÁ Yot-Domínguez C Conde-Jiménez J Ramírez Martín P . The influence of emotional burnout and resilience on the psychological distress of nursing students during the COVID-19 pandemic. Int J Ment Health Nurs. (2022) 31:1457–66. doi: 10.1111/inm.13046 35938942 PMC9538541

[49] Abram MD Jacobowitz W . Resilience and burnout in healthcare students and inpatient psychiatric nurses: a between-groups study of two populations. Arch Psychiatr Nurs. (2021) 35:1–8. doi: 10.1016/j.apnu.2020.10.008 33593500 PMC7577217

[50] Ríos-Rísquez MI García-Izquierdo M Sabuco-Tebar EA Carrillo-García C Solano-Ruiz C . Connections between academic burnout, resilience, and psychological well-being in nursing students: a longitudinal study. J Adv Nurs. (2018) 74:2777–84. doi: 10.1111/jan.13794 29992596

[51] Zhang Y Guan C Jiang J Zhu C Hu X . Mediating effect of resilience on the relationship between perceived social support and burnout among Chinese palliative nurses. J Clin Nurs. (2023) 32:3887–97. doi: 10.1111/jocn.16532 36101490

[52] Alkrenawi Y Haddad G Haj Yahya S Rozani V . Post-traumatic growth in nursing students: examining resilience, secondary stress, burnout, and satisfaction in clinical practice. Nurse Educ. (2025) 50:189–94. doi: 10.1097/nne.0000000000001866 40184490 PMC12594122

[53] Kolutek R Erkutlu H Chafra J . Workplace violence and nurses’ psychological well-being: the mediating role of burnout and the moderating role of psychological resilience. Arch Psychiatr Nurs. (2024) 53:177–83. doi: 10.1016/j.apnu.2024.10.015 39615933

[54] LaPlante RD Patricia RP Jurgens CY . Professional fulfillment, psychological safety, and resilience in relation to burnout among critical care nurses. Dimens Crit Care Nurs. (2026) 45:141–8. doi: 10.1097/dcc.0000000000000755 41921019

[55] Zhang X Jiang X Ni P Li H Li C Zhou Q . Association between resilience and burnout of front-line nurses at the peak of the COVID-19 pandemic: positive and negative affect as mediators in Wuhan. Int J Ment Health Nurs. (2021) 30:939–54. doi: 10.1111/inm.12847 33893718 PMC8251287

[56] Arrogante Maroto Ó Aparicio-Zaldívar EG . Tools to face burnout in nursing: social support, resilience and coping strategies. Rev Enferm. (2017). 30277359

[57] Guo YF Plummer V Lam L Wang Y Cross W Zhang JP . The effects of resilience and turnover intention on nurses’ burnout: findings from a comparative cross-sectional study. J Clin Nurs. (2019) 28:499–508. doi: 10.1111/jocn.14637 30091259

[58] Alkubati SA Alshammari E Albaqawi H Alharbi A Laradhi AO Albani GF . The mediating effect of resilience on the relationship between alarm fatigue and burnout among critical care nurses. Aust Crit Care. (2025) 38:101258. doi: 10.1016/j.aucc.2025.101258 40412257

[59] Min D . Effects of resilience, burnout, and work-related physical pain on work-life balance of registered nurses in South Korean nursing homes: a cross-sectional study. Med (Baltim). (2022) 101:e29889. doi: 10.1097/md.0000000000029889 35905217 PMC9333544

[60] Ogińska-Bulik N Michalska P . Psychological resilience and secondary traumatic stress in nurses working with terminally ill patients-the mediating role of job burnout. Psychol Serv. (2021) 18:398–405. doi: 10.1037/ser0000421 32091233

[61] García-Izquierdo M Meseguer de Pedro M Ríos-Risquez MI Sánchez MIS . Resilience as a moderator of psychological health in situations of chronic stress (burnout) in a sample of hospital nurses. J Nurs Sch. (2017) 50:228–36. doi: 10.1111/jnu.12367 29266720

[62] Jiménez-García S Lázaro E Vizcaya-Moreno F . Resilience as a mediator between burnout and health among nurses during the COVID-19 pandemic: a cross-sectional survey in late 2021. Nurs Open. (2026) 13:e70526. doi: 10.1002/nop2.70526 41981743 PMC13079419

[63] Gensimore MM Maduro RS Morgan MK McGee GW Zimbro KS . The effect of nurse practice environment on retention and quality of care via burnout, work characteristics, and resilience: a moderated mediation model. J Nurs Adm. (2020) 50:546–53. doi: 10.1097/nna.0000000000000932 32925666

[64] Ríos-Rísquez MI García-Izquierdo M Sabuco-Tebar EA Carrillo-García C Martínez-Roche ME . An exploratory study of the relationship between resilience, academic burnout and psychological health in nursing students. Contemp Nurse. (2016) 52:430–9. doi: 10.1080/10376178.2016.1213648 27436758

[65] Cha YJ Lee KS Cho JH Choi IS Lee D . Effect of job stress on burnout among nurses responding to COVID-19: the mediating effect of resilience. Int J Env Res Public Health. (2022) 19:5409. doi: 10.3390/ijerph19095409 35564803 PMC9104873

[66] Jeong YJ Shin S . The relationship between secondary traumatic stress and burnout in critical care nurses: the mediating effect of resilience. Intensive Crit Care Nurs. (2023) 74:103327. doi: 10.1016/j.iccn.2022.103327 36208974

[67] Vieira LS MaChado WL Dal Pai D Magnago TS . Burnout and resilience in intensive care nursing professionals in the face of COVID-19: a multicenter study. Rev Lat Am Enferm. (2022) 30:e3589. doi: 10.1590/1518-8345.5778.3589 35649092 PMC9150431

[68] Sikaras C Pachi A Alikanioti S Ilias I Sideri EP Tselebis A . Occupational burnout and insomnia in relation to psychological resilience among greek nurses in the post-pandemic era. Behav Sci (Basel). (2025) 15:126. doi: 10.3390/bs15020126 40001757 PMC11851848

[69] Jose S Dhandapani M Cyriac MC . Burnout and resilience among frontline nurses during COVID-19 pandemic: a cross-sectional study in the emergency department of a tertiary care center, north India. Indian J Crit Care Med. (2020) 24:1081–8. doi: 10.5005/jp-journals-10071-23667 33384515 PMC7751034

[70] Zahednezhad H Zareiyan A Zargar Balaye Jame S . Relationship between quality of work-life, resilience and burnout among nursing professionals during COVID-19 pandemic in Iran: a cross-sectional study. Belitung Nurs J. (2021) 7:508–15. doi: 10.33546/bnj.1702 37497286 PMC10367986

[71] Souza-Costa AC Oliveira HC Guirardello EB . Relationship among workplace bullying, job satisfaction, burnout, and turnover intention: the mediating role of nurses’ resilience. Nurs Res Pr. (2026) 2026:8868659. doi: 10.1155/nrp/8868659 42038944 PMC13107538

[72] Rees CS Heritage B Osseiran-Moisson R Chamberlain D Cusack L Anderson J . Can we predict burnout among student nurses? An exploration of the ICWR-1 model of individual psychological resilience. Front Psychol. (2016) 7:1072. doi: 10.3389/fpsyg.2016.01072 27486419 PMC4949488

[73] Lee EY Kim KJ Ko S Song EK . Communication competence and resilience are modifiable factors for burnout of operating room nurses in South Korea. BMC Nurs. (2022) 21:203. doi: 10.1186/s12912-022-00985-0 35897026 PMC9327268

[74] Jamebozorgi MH Karamoozian A Bardsiri TI Sheikhbardsiri H . Nurses burnout, resilience, and its association with socio-demographic factors during COVID-19 pandemic. Front Psychiatry. (2021) 12:803506. doi: 10.3389/fpsyt.2021.803506 35095618 PMC8795765

[75] Kutluturkan S Sozeri E Uysal N Bay F . Resilience and burnout status among nurses working in oncology. Ann Gen Psychiatry. (2016) 15:33. doi: 10.1186/s12991-016-0121-3 27895699 PMC5109709

[76] Kchaou A Kotti N Dhouib F Hrairi A Ben Messaoud W Trigui A . Promoting resilience to prevent burnout and secondary traumatic stress among intensive care units’ nurses. J Intensive Care Soc. (2025) 26:479–84. doi: 10.1177/17511437251357121 40933819 PMC12417468

[77] Baugh A Reiser V Zhao J Klein SJ Rosenzweig MQ . Burnout and resiliency among advanced practice providers in oncology care. J Adv Pr Oncol. (2024) 15:95–101. doi: 10.6004/jadpro.2024.15.2.2 39132553 PMC11308540

[78] Hwang S Lee J . The influence of COVID-19-related resilience on depression, job stress, sleep quality, and burnout among intensive care unit nurses. Front Psychol. (2023) 14:1168243. doi: 10.3389/fpsyg.2023.1168243 37205066 PMC10186099

[79] Shalom T Ogni L Freud T Bashkin O . Burnout, anxiety, and resilience among nurses during wartime: evidence from a cross-sectional study. BMC Nurs. (2025) 24:1424. doi: 10.1186/s12912-025-04061-1 41272679 PMC12639681

[80] Miyazaki A Sankai T Omiya T . Experience and resilience of Japanese public health nurses during the COVID-19 pandemic and their impact on burnout. Healthc (Basel). (2023) 11:1114. doi: 10.3390/healthcare11081114 37107949 PMC10137901

[81] Altharman HA Alnaqi RI Buanz SF Alsenayien AY Siraj RA . Exploring the relationship between burnout, resilience, and dropout intention among nursing students during clinical training in Saudi Arabia. SAGE Open Nurs. (2023) 9:23779608231210084. doi: 10.1177/23779608231210084 37916033 PMC10617278

[82] Suazo Galdames I Molero Jurado MDM Fernández Martínez E Pérez-Fuentes M . Resilience, burnout and mental health in nurses: a latent mediation model. J Clin Med. (2024) 13:2769. doi: 10.3390/jcm13102769 38792311 PMC11121760

[83] Lee EJ Sung MH Ahn HK Kim YA . Effect of incivility, resilience, and social support experienced by nursing students on burnout in clinical practice. Korean J Women Health Nurs. (2019) 25:86. doi: 10.4069/kjwhn.2019.25.1.86 37679932

[84] Alia A Sandi YDL Agustina HS Andarini E Rosyda R . An investigation of the association between stress levels, perfectionism, academic motivation, and resilience with academic burnout among nursing students: a cross-sectional study. Psychiatria. (2025) 22:49–56. doi: 10.5603/psych.107134

[85] Buljubašić A Gizdic A Drmić S Murn L Antičević V . Psychological resilience and life satisfaction: effects of burnout and post-traumatic stress in the working environment of the COVID-19 pandemic. Soc Psihijatr Zadovoljstvo Životom: Učin Sagorijevanja Posttraumatskog Stresa U Radn Okruž Pandemije COVID-19. (2023) 51:183–201. doi: 10.24869/spsih.2023.183

[86] Zanjani Z Joekar S Omidi A . The mental health and job burnout of nurses during the COVID-19 outbreak: resilience as a mediator. Iran J Psychiatry Behav Sci. (2021) 15. doi: 10.5812/ijpbs.109925

[87] Mohammadi F Bijani M Borzou SR Oshvandi K Khazaei S Bashirian S . Resilience, occupational burnout, and parenting stress in nurses caring for COVID-19 patients. Neuropsychiatr Neuropsychol. (2021) 16:116–23. doi: 10.5114/nan.2021.113311

[88] Aghaei HA Asadi ZS . The influence of organizational culture on resilience by mediatory effects of occupational stress, job satisfaction, and burnout in nurses: structural equation modeling. Iran Red Crescent Med J. (2020) 22. doi: 10.5812/ircmj.102332

[89] Ríos-Rísquez MI Carrillo-García C Sabuco-Tebar EA . Resilience and burnout syndrome in nursing students and its relationship with sociodemographic variables and interpersonal relationship. Int J Psychol Res. (2012) 5:88–95. doi: 10.21500/20112084.765

[90] Ghoneam M El-Ashry A Sabek E Seweid M . Influence of clinical practice stressors on critical care nursing student’s burnout: resilience as a mediating factor. Teach Learn Nurs. (2025) 20:e1136–44. doi: 10.1016/j.teln.2025.05.03142574925

[91] Rubaca U Munir M . Supervisor incivility, emotional exhaustion and nurses’ job neglect: the moderating role of resilience and professional calling. Int J Organ Theory Behav. (2025) 28:214–33. doi: 10.1108/ijotb-01-2023-002935579975

[92] Ata E Yilmaz E Bayrak N . Resilience, occupational satisfaction, burnout and compassion fatigue in Turkish intensive care nurses. Kontakt-J Nurs Soc Sci Relat Health Illn. (2020) 22:152–8. doi: 10.32725/kont.2020.028

[93] Kim H Kim H Yeun E . Impact of resilience, emotional exhaustion, and communication competency on organizational commitment among nurses in a tertiary hospital setting in South Korea: a cross-sectional study. SAGE Open. (2025) 15:21582440251385661. doi: 10.1177/21582440251385661

[94] Ertem M Capa S Karakas M Ensari H Koc A . Investigation of the relationship between nurses’ burnout and psychological resilience levels. Clin Exp Health Sci. (2020) 10:9–15. doi: 10.33808/clinexphealthsci.600924

[95] Alonazi O Alshowkan A Shdaifat E . Exploring the relationships between compassion satisfaction, burnout, secondary traumatic stress, and resilience among nurses: a structural equation modeling approach. Nurs Forum (Auckl). (2025) 2025:5638025. doi: 10.1155/nuf/5638025

[96] Ko E Kim H Kim G Kim R So H . Burnout amongst nurses in cancer wards: impact of nursing professionalism, perfectionism, and resilience. Asian Oncol Nurs. (2018) 18:214. doi: 10.5388/aon.2018.18.4.214

[97] Orgambídez A . Relationship between psychological capital and nursing burnout: a systematic review and meta-analysis. Int Nurs Rev. (2025) 72:e13072. doi: 10.1111/inr.13072 39569557 PMC12206688

[98] Dai X Xie C Wu Y Chen T Lu F . Factors associated with burnout among Chinese operating room nurses: a meta-analysis. BMC Nurs. (2025) 24:312. doi: 10.1186/s12912-025-02914-3 40133979 PMC11934704

[99] Vanhove AJ Herian MN Perez ALU Harms PD Lester PB . Can resilience be developed at work? A meta-analytic review of resilience-building programme effectiveness. J Occup Organ Psychol. (2016) 89:278–307. doi: 10.1111/joop.1212340046247

[100] Robertson IT Cooper CL Sarkar M Curran T . Resilience training in the workplace from 2003 to 2014: a systematic review. J Occup Organ Psychol. (2015) 88:533–62. doi: 10.1111/joop.1212040046247

[101] Hobfoll SE . Conservation of resources: a new attempt at conceptualizing stress. Am Psychol. (1989) 44:513–24. doi: 10.1037/0003-066X.44.3.513 2648906

[102] Bakker AB Demerouti E . The job demands-resources model: State of the art. J Manag Psychol. (2007) 22:309–28. doi: 10.1108/0268394071073311535579975

