# Supplementary material for: Psychological resilience and burnout in nursing interns versus registered nurses: a systematic review and meta-analysis
Source: Front Psychiatry. 2026 Oct 14;17:1920134. doi: 10.3389/fpsyt.2026.1920134 (PMC13714640; doi:10.3389/fpsyt.2026.1920134)
Supplement: Supplementary file 1 [file Table1.docx]

# Supplementary Appendix A. Search strategies and results across the nine databases

*Supplementary Table. Search strategies and yields across nine databases*
Search concepts: psychological resilience × nurses × burnout. Study design was applied at full-text screening and was not built into the search strings.PROSPERO registration: CRD420261427008 | Search date: 2026-06-20

**1. Records retrieved per database**

| **No.** | **Database** | **Field / syntax** | **Records** | **Search date** |
| --- | --- | --- | --- | --- |
| 1 | PubMed | MeSH + [tiab] | 1236 | 2026-06-20 |
| 2 | Embase | Emtree/exp + :ti,ab | 1039 | 2026-06-20 |
| 3 | CINAHL | MH + TI/AB | 1428 | 2026-06-20 |
| 4 | Web of Science | TS=(topic) | 2087 | 2026-06-20 |
| 5 | Cochrane Library | MeSH + :ti,ab,kw | 147 | 2026-06-20 |
| 6 | CNKI | SU=(topic) | 127 | 2026-06-20 |
| 7 | Wanfang Data | Topic field | 364 | 2026-06-20 |
| 8 | VIP (CQVIP) | M=(title/keyword) | 130 | 2026-06-20 |
| 9 | SinoMed | Smart search | 328 | 2026-06-20 |
|  | Total (before de-duplication) | — | **6886** | 2026-06-20 |

**2. Full search strings by database**

**1. PubMed (1236 records)**

("Resilience, Psychological"[Mesh] OR resilien*[tiab] OR "psychological resilience"[tiab] OR "ego resilience"[tiab] OR hardiness[tiab]) AND ("Nurses"[Mesh] OR "Students, Nursing"[Mesh] OR "Nursing Staff"[Mesh] OR nurse[tiab] OR nurses[tiab] OR "nursing staff"[tiab] OR "nursing student*"[tiab] OR "student nurse*"[tiab] OR "nursing intern*"[tiab] OR "nursing undergraduate*"[tiab] OR "newly graduated nurse*"[tiab] OR "new graduate nurse*"[tiab] OR "new nurse*"[tiab] OR "registered nurse*"[tiab]) AND ("Burnout, Psychological"[Mesh] OR "Burnout, Professional"[Mesh] OR burnout[tiab] OR "burn out"[tiab] OR "burn-out"[tiab] OR "emotional exhaustion"[tiab] OR depersonali*[tiab] OR "compassion fatigue"[tiab] OR "job burnout"[tiab] OR "occupational burnout"[tiab])

**2. Embase (1039 records)**

('resilience'/exp OR resilien*:ti,ab OR 'ego resilience':ti,ab OR hardiness:ti,ab) AND ('nurse'/exp OR 'nursing student'/exp OR nurse:ti,ab OR nurses:ti,ab OR 'nursing staff':ti,ab OR 'nursing student*':ti,ab OR 'student nurse*':ti,ab OR 'nursing intern*':ti,ab OR 'newly graduated nurse*':ti,ab OR 'new nurse*':ti,ab OR 'registered nurse*':ti,ab) AND ('burnout'/exp OR burnout:ti,ab OR 'burn out':ti,ab OR 'emotional exhaustion':ti,ab OR depersonali*:ti,ab OR 'compassion fatigue':ti,ab OR 'job burnout':ti,ab)

**3. CINAHL (1428 records)**

( (MH "Hardiness") OR (MH "Resilience, Psychological") OR TI (resilien* OR hardiness OR "ego resilience") OR AB (resilien* OR hardiness OR "ego resilience") ) AND ( (MH "Nurses+") OR (MH "Students, Nursing+") OR (MH "Nursing Staff, Hospital+") OR TI (nurse* OR "nursing student*" OR "student nurse*" OR "nursing intern*" OR "newly graduated nurse*" OR "new nurse*" OR "registered nurse*") OR AB (nurse* OR "nursing student*" OR "student nurse*" OR "nursing intern*" OR "newly graduated nurse*" OR "new nurse*" OR "registered nurse*") ) AND ( (MH "Burnout, Professional+") OR TI (burnout OR "burn out" OR "emotional exhaustion" OR depersonali* OR "compassion fatigue" OR "job burnout") OR AB (burnout OR "burn out" OR "emotional exhaustion" OR depersonali* OR "compassion fatigue" OR "job burnout") )

**4. Web of Science (2087 records)**

TS=(resilien* OR hardiness OR "ego resilience") AND TS=(nurse OR nurses OR "nursing staff" OR "nursing student*" OR "student nurse*" OR "nursing intern*" OR "newly graduated nurse*" OR "new nurse*" OR "registered nurse*") AND TS=(burnout OR "burn out" OR "emotional exhaustion" OR depersonali* OR "compassion fatigue" OR "job burnout")

**5. Cochrane Library (147 records)**

#1 MeSH descriptor: [Nurses] explode all trees
#2 MeSH descriptor: [Students, Nursing] explode all trees
#3 MeSH descriptor: [Nursing Staff] explode all trees
#4 MeSH descriptor: [Education, Nursing] explode all trees
#5 (nurse* OR (nursing NEXT student*) OR (student NEXT nurse*) OR "nursing staff" OR "nurse education" OR (undergraduate NEAR/3 nurs*) OR (nursing NEXT trainee*)):ti,ab,kw
#6 #1 OR #2 OR #3 OR #4 OR #5
#7 MeSH descriptor: [Resilience, Psychological] explode all trees
#8 (resilien* OR "psychological resilience" OR hardiness OR "mental toughness" OR "ego-resilience" OR "adaptive capacity"):ti,ab,kw
#9 #7 OR #8
#10 MeSH descriptor: [Burnout, Psychological] explode all trees
#11 MeSH descriptor: [Burnout, Professional] explode all trees
#12 (burnout OR "burn out" OR "burn-out" OR "emotional exhaustion" OR depersonali* OR "compassion fatigue" OR "occupational stress" OR "job stress" OR "work-related stress"):ti,ab,kw
#13 #10 OR #11 OR #12
#14 #6 AND #9 AND #13

**6. CNKI (China National Knowledge Infrastructure) (127 records)**

SU=('护士'+'护理人员'+'护生'+'护理学生'+'护理本科生'+'实习护士'+'规培护士'+'注册护士'+'临床护士') AND SU=('心理韧性'+'心理弹性'+'韧性'+'复原力'+'心理复原力'+'抗逆力'+'坚韧性'+'心理坚韧') AND SU=('职业倦怠'+'工作倦怠'+'倦怠'+'职业疲溃'+'情感耗竭'+'情绪耗竭'+'去人格化'+'共情疲劳'+'同情疲劳'+'职业枯竭')

**7. Wanfang Data (364 records)**

主题:("护士" OR "护生" OR "护理人员" OR "护理学生" OR "实习护士") AND 主题:("心理韧性" OR "心理弹性" OR "韧性" OR "复原力" OR "抗逆力" OR "坚韧性") AND 主题:("职业倦怠" OR "工作倦怠" OR "倦怠" OR "情感耗竭" OR "情绪耗竭" OR "共情疲劳" OR "同情疲劳")

**8. VIP (CQVIP) (130 records)**

M=(护士 OR 护生 OR 护理人员 OR 护理学生 OR 实习护士) AND M=(心理韧性 OR 心理弹性 OR 韧性 OR 复原力 OR 抗逆力 OR 坚韧性) AND M=(职业倦怠 OR 工作倦怠 OR 倦怠 OR 情感耗竭 OR 情绪耗竭 OR 共情疲劳 OR 同情疲劳)

**9. SinoMed (Chinese Biomedical Literature Database) (328 records)**

#1 护士 OR 护生 OR 护理人员 OR 护理学生 OR 实习护士
#2 心理韧性 OR 心理弹性 OR 韧性 OR 复原力 OR 抗逆力 OR 坚韧性
#3 职业倦怠 OR 工作倦怠 OR 倦怠 OR 情感耗竭 OR 情绪耗竭 OR 共情疲劳 OR 同情疲劳
#4 #1 AND #2 AND #3

**Notes. (1) The same three-concept structure (psychological resilience × nurses × burnout) was used across all nine databases; synonyms were adapted to each database controlled vocabulary (MeSH / Emtree / CINAHL Subject Headings) and syntax, following PRISMA-S. (2) In the English databases (PubMed, Embase, CINAHL, Web of Science), "psychological capital" was excluded throughout; the operational definition of resilience included the closely related constructs resilience, hardiness, and ego-resilience, keeping the Chinese and English searches aligned. (3) Study design (cross-sectional / correlational) was applied at full-text screening rather than in the search strings, to preserve sensitivity. (4) The nine databases yielded 6886 records before de-duplication, which were imported into reference-management software for de-duplication before screening.**
