# Supplementary material for: Psychological resilience and burnout in nursing interns versus registered nurses: a systematic review and meta-analysis
Source: Front Psychiatry. 2026 Oct 14;17:1920134. doi: 10.3389/fpsyt.2026.1920134 (PMC13714640; doi:10.3389/fpsyt.2026.1920134)
Supplement: Supplementary file 2 [file Table2.docx]

# **Supplementary Table S1. AHRQ item-level quality appraisal of included studies**

| **Study** | **Item 1** | **Item 2** | **Item 3** | **Item 4** | **Item 5** | **Item 6** | **Item 7** | **Item 8** | **Item 9** | **Item 10** | **Item 11** | **Total** | **Quality** |
| --- | --- | --- | --- | --- | --- | --- | --- | --- | --- | --- | --- | --- | --- |
| Gao (2025) (14) | 1 | 1 | 1 | 0 | 0 | 1 | 1 | 1 | 0 | 1 | 0 | 7 | Moderate |
| Chai (2025)(15) | 1 | 1 | 1 | 1 | 0 | 1 | 0 | 1 | 1 | 1 | 0 | 8 | High |
| Yun (2022)(16) | 1 | 0 | 1 | 0 | 0 | 0 | 0 | 1 | 0 | 1 | 0 | 4 | Low |
| Gao (2023) (17) | 1 | 1 | 1 | 0 | 0 | 1 | 1 | 1 | 0 | 1 | 0 | 7 | Moderate |
| Huang (2025) (18) | 1 | 1 | 1 | 0 | 0 | 1 | 0 | 1 | 0 | 1 | 0 | 6 | Moderate |
| Yan (2024) (19) | 1 | 1 | 1 | 0 | 0 | 1 | 0 | 1 | 0 | 0 | 0 | 5 | Low |
| Lyu (2023) (20) | 1 | 1 | 1 | 0 | 0 | 1 | 1 | 1 | 0 | 1 | 0 | 7 | Moderate |
| Yang (2023)(21) | 1 | 1 | 1 | 1 | 0 | 1 | 1 | 1 | 0 | 1 | 0 | 8 | High |
| Guo JB (2019) (22) | 1 | 1 | 1 | 0 | 0 | 1 | 1 | 1 | 0 | 1 | 0 | 7 | Moderate |
| Sun YX (2019)(23) | 1 | 1 | 1 | 0 | 0 | 1 | 1 | 1 | 1 | 1 | 0 | 8 | High |
| Sun YZ (2016)(24) | 1 | 1 | 1 | 0 | 0 | 1 | 1 | 1 | 1 | 1 | 0 | 8 | High |
| Ke (2024)(25) | 1 | 1 | 1 | 0 | 0 | 1 | 1 | 1 | 0 | 1 | 0 | 7 | Moderate |
| Chen (2024)(26) | 1 | 1 | 1 | 0 | 0 | 1 | 1 | 1 | 0 | 1 | 0 | 7 | Moderate |
| Cui (2019) (27) | 1 | 0 | 0 | 0 | 0 | 1 | 1 | 0 | 1 | 1 | 0 | 5 | Low |
| Zou (2014) (28) | 1 | 0 | 0 | 0 | 0 | 1 | 1 | 1 | 1 | 1 | 0 | 6 | Moderate |
| He (2009)(29) | 1 | 1 | 0 | 0 | 0 | 1 | 1 | 0 | 1 | 1 | 0 | 6 | Moderate |
| Ou (2024)(30) | 1 | 1 | 1 | 0 | 0 | 1 | 1 | 1 | 1 | 1 | 0 | 8 | High |
| Huang ZX (2019)(31) | 1 | 1 | 0 | 0 | 0 | 1 | 1 | 1 | 1 | 1 | 0 | 7 | Moderate |
| Zhou (2020)(32) | 1 | 1 | 1 | 0 | 0 | 1 | 1 | 1 | 0 | 1 | 0 | 7 | Moderate |
| Li (2015)(33) | 1 | 0 | 0 | 0 | 0 | 1 | 1 | 1 | 0 | 1 | 0 | 5 | Low |
| Al-Harrasi (2024)(34) | 1 | 1 | 1 | 0 | 0 | 1 | 1 | 1 | 0 | 1 | 0 | 7 | Moderate |
| Georges (2022)(35) | 1 | 1 | 1 | 0 | 0 | 1 | 1 | 0 | 0 | 0 | 0 | 5 | Low |
| Yildirim (2024)(36) | 1 | 1 | 1 | 0 | 0 | 1 | 0 | 0 | 0 | 1 | 0 | 5 | Low |
| Guo (2018)(37) | 1 | 1 | 1 | 0 | 0 | 1 | 0 | 1 | 0 | 1 | 0 | 6 | Moderate |
| Lang (2021)(38) | 1 | 1 | 1 | 0 | 0 | 1 | 1 | 1 | 1 | 1 | 0 | 8 | High |
| Rushton (2015)(39) | 1 | 0 | 0 | 0 | 0 | 1 | 0 | 1 | 0 | 1 | 0 | 4 | Low |
| Luo (2022) (40) | 1 | 1 | 1 | 1 | 0 | 1 | 1 | 1 | 1 | 1 | 0 | 9 | High |
| Yang (2018)(41) | 1 | 1 | 1 | 0 | 0 | 1 | 1 | 1 | 0 | 1 | 0 | 7 | Moderate |
| Lee (2025)(42) | 1 | 1 | 1 | 0 | 0 | 1 | 0 | 1 | 0 | 0 | 0 | 5 | Low |
| Rivas (2021)(43) | 1 | 1 | 1 | 0 | 0 | 1 | 0 | 0 | 0 | 0 | 0 | 4 | Low |
| Zhang (2024)‡(44) | 1 | 1 | 1 | 0 | 0 | 1 | 1 | 1 | 0 | 1 | 0 | 7 | Moderate |
| Jiménez-Fernández (2022) (45) | 1 | 1 | 1 | 0 | 0 | 1 | 0 | 1 | 0 | 0 | 0 | 5 | Low |
| Abdulmohdi (2024)(46) | 1 | 1 | 1 | 0 | 0 | 1 | 0 | 1 | 0 | 1 | 0 | 6 | Moderate |
| Ochs (2024)(47) | 1 | 0 | 0 | 0 | 0 | 1 | 0 | 0 | 0 | 1 | 0 | 3 | Low |
| Merino-Godoy (2022)(48) | 1 | 0 | 1 | 0 | 0 | 1 | 0 | 0 | 0 | 0 | 0 | 3 | Low |
| Abram (2021)(49) | 1 | 0 | 1 | 0 | 0 | 1 | 1 | 1 | 1 | 0 | 0 | 6 | Moderate |
| Ríos-Risquez (2018)‡ (50) | 1 | 1 | 1 | 0 | 0 | 1 | 0 | 1 | 0 | 1 | 1 | 7 | Moderate |
| Zhang (2023)(51) | 1 | 1 | 1 | 1 | 0 | 1 | 1 | 1 | 1 | 0 | 0 | 8 | High |
| Alkrenawi (2025)(52) | 1 | 1 | 1 | 0 | 0 | 1 | 1 | 1 | 0 | 1 | 0 | 7 | Moderate |
| Kolutek (2024)(53) | 1 | 1 | 1 | 1 | 0 | 1 | 0 | 1 | 1 | 1 | 0 | 8 | High |
| LaPlante (2026)(54) | 1 | 1 | 1 | 0 | 0 | 1 | 1 | 0 | 1 | 1 | 0 | 7 | Moderate |
| Zhang (2021)(55) | 1 | 0 | 1 | 0 | 0 | 1 | 1 | 0 | 0 | 1 | 0 | 5 | Low |
| Arrogante Maroto (2017) (56) | 1 | 1 | 1 | 0 | 0 | 1 | 1 | 1 | 0 | 1 | 0 | 7 | Moderate |
| Guo (2019) (57) | 1 | 1 | 1 | 0 | 0 | 1 | 1 | 1 | 0 | 1 | 0 | 7 | Moderate |
| Alkubati (2025)(58) | 1 | 1 | 1 | 1 | 0 | 1 | 1 | 1 | 0 | 1 | 0 | 8 | High |
| Min (2022) (59) | 1 | 1 | 1 | 0 | 0 | 1 | 0 | 1 | 0 | 0 | 0 | 5 | Low |
| Ogińska-Bulik (2021)(60) | 1 | 1 | 1 | 0 | 0 | 1 | 1 | 1 | 0 | 1 | 0 | 7 | Moderate |
| García-Izquierdo (2017)(61) | 1 | 0 | 0 | 0 | 0 | 1 | 0 | 1 | 0 | 1 | 0 | 4 | Low |
| Jiménez-García (2026)(62) | 1 | 1 | 1 | 0 | 0 | 1 | 0 | 1 | 0 | 0 | 0 | 5 | Low |
| Gensimore (2020) (63) | 1 | 1 | 0 | 0 | 0 | 1 | 1 | 1 | 1 | 0 | 0 | 6 | Moderate |
| Ríos-Risquez (2016)‡ (64) | 1 | 1 | 1 | 0 | 0 | 1 | 1 | 1 | 0 | 1 | 0 | 7 | Moderate |
| Cha (2022) (65) | 1 | 0 | 1 | 0 | 0 | 1 | 1 | 1 | 0 | 1 | 0 | 6 | Moderate |
| Jeong (2023)(66) | 1 | 1 | 1 | 0 | 0 | 1 | 1 | 0 | 0 | 1 | 0 | 6 | Moderate |
| Vieira (2022)(67) | 1 | 1 | 1 | 0 | 0 | 1 | 0 | 1 | 0 | 0 | 0 | 5 | Low |
| Sikaras (2025)(68) | 1 | 1 | 1 | 0 | 0 | 1 | 0 | 1 | 0 | 1 | 0 | 6 | Moderate |
| Jose (2020)(69) | 1 | 0 | 1 | 1 | 0 | 1 | 0 | 0 | 0 | 1 | 0 | 5 | Low |
| Zahednezhad (2021)(70) | 1 | 1 | 1 | 0 | 0 | 1 | 0 | 0 | 1 | 1 | 0 | 6 | Moderate |
| Souza-Costa (2026)(71) | 1 | 1 | 1 | 0 | 0 | 1 | 0 | 1 | 0 | 0 | 0 | 5 | Low |
| Rees (2016) (72) | 1 | 1 | 1 | 0 | 0 | 1 | 1 | 1 | 1 | 0 | 0 | 7 | Moderate |
| Lee (2022)(73) | 1 | 1 | 1 | 0 | 0 | 1 | 1 | 1 | 0 | 1 | 0 | 7 | Moderate |
| Jamebozorgi (2021) (74) | 1 | 1 | 0 | 0 | 0 | 1 | 0 | 1 | 0 | 1 | 0 | 5 | Low |
| Kutluturkan (2016)(75) | 1 | 1 | 0 | 0 | 0 | 1 | 1 | 1 | 0 | 1 | 0 | 6 | Moderate |
| Kchaou (2025)(76) | 1 | 1 | 1 | 0 | 0 | 1 | 1 | 1 | 0 | 1 | 0 | 7 | Moderate |
| Baugh (2024)(77) | 1 | 1 | 1 | 0 | 0 | 0 | 0 | 0 | 0 | 1 | 0 | 4 | Low |
| Hwang (2023) (78) | 1 | 1 | 1 | 0 | 0 | 1 | 1 | 1 | 1 | 1 | 0 | 8 | High |
| Shalom (2025)(79) | 1 | 1 | 1 | 0 | 0 | 1 | 0 | 1 | 0 | 1 | 0 | 6 | Moderate |
| Miyazaki (2023)(80) | 1 | 1 | 1 | 0 | 0 | 1 | 0 | 1 | 0 | 1 | 0 | 6 | Moderate |
| Altharman (2023) (81) | 1 | 1 | 1 | 0 | 0 | 1 | 1 | 0 | 0 | 0 | 0 | 5 | Low |
| Suazo Galdames (2024)(82) | 1 | 0 | 1 | 1 | 0 | 1 | 1 | 0 | 0 | 0 | 0 | 5 | Low |
| Lee (2019) (83) | 1 | 1 | 1 | 0 | 0 | 1 | 0 | 0 | 1 | 1 | 0 | 6 | Moderate |
| Alia (2025)‡ (84) | 1 | 1 | 1 | 0 | 0 | 1 | 1 | 0 | 0 | 1 | 0 | 6 | Moderate |
| Buljubašić (2023)(85) | 1 | 0 | 1 | 0 | 0 | 1 | 0 | 1 | 1 | 0 | 0 | 5 | Low |
| Zanjani (2021) (86) | 1 | 1 | 1 | 0 | 0 | 1 | 0 | 1 | 0 | 0 | 0 | 5 | Low |
| Mohammadi (2021) (87) | 1 | 1 | 1 | 0 | 0 | 1 | 1 | 1 | 0 | 1 | 0 | 7 | Moderate |
| Aghaei (2020) (88) | 1 | 1 | 1 | 1 | 0 | 1 | 1 | 1 | 1 | 1 | 0 | 9 | High |
| Rísquez (2012)‡ (89) | 1 | 1 | 1 | 0 | 0 | 1 | 1 | 0 | 0 | 1 | 0 | 6 | Moderate |
| Ghoneam (2025)(90) | 1 | 1 | 1 | 0 | 0 | 1 | 1 | 0 | 1 | 1 | 0 | 7 | Moderate |
| Rubaca (2025) (91) | 1 | 1 | 1 | 0 | 0 | 1 | 1 | 1 | 0 | 1 | 0 | 7 | Moderate |
| Ata (2020) (92) | 1 | 1 | 1 | 0 | 0 | 1 | 1 | 0 | 0 | 1 | 0 | 6 | Moderate |
| Kim (2025)(93) | 1 | 1 | 1 | 0 | 0 | 1 | 1 | 1 | 1 | 1 | 0 | 8 | High |
| Ertem (2020)(94) | 1 | 1 | 1 | 0 | 0 | 1 | 0 | 0 | 0 | 0 | 0 | 4 | Low |
| Alonazi (2025) (95) | 1 | 1 | 1 | 0 | 0 | 1 | 1 | 0 | 0 | 0 | 0 | 5 | Low |
| Ko (2018)(96) | 1 | 1 | 1 | 0 | 0 | 1 | 1 | 1 | 0 | 1 | 0 | 7 | Moderate |

AHRQ 11-item checklist items: 1, source of information defined; 2, inclusion/exclusion criteria listed or referenced; 3, time period for identifying subjects indicated; 4, subjects consecutive if not population-based; 5, evaluators of subjective components masked; 6, quality-assurance assessments described; 7, patient exclusions from analysis explained; 8, confounding assessed and/or controlled; 9, handling of missing data explained; 10, response rate and completeness of data collection summarised; 11, expected follow-up and proportion with incomplete data/follow-up clarified.

*Note.* Each item is scored 1 (criterion met) or 0 (not met or not reported); Total = sum of the 11 items. Quality grade follows the registered thresholds: High ≥8, Moderate 6–7, Low ≤5. Bracketed numbers are reference citations and match main-text Table 1 and the reference list; studies are listed in the same order as Table 1. ‡ academic-burnout study (nursing students), reported separately and not included in the 78-study occupational-burnout analysis.

# **Supplementary Table S2. Group × burnout-construct cross-tabulation**

| **Group** | **Academic (student) burnout** | **Occupational burnout** | **Total** |  |  |  |  |  |
| --- | --- | --- | --- | --- | --- | --- | --- | --- |
| A — Nursing interns | 5 | 6 | 11 |  |  |  |  |  |
| B — Registered nurses | 0 | 72 | 72 |  |  |  |  |  |
| Total | 5 | 78 | 83 |  |  |  |  |  |
| Group | Occupational MBI family | Student/academic MBI (MBI-SS) | CBI | ProQOL | OLBI/OBI | Single-item EE | Other | Total |
| A — Interns | 3 | 2 | 1 | 2 | 0 | 1 | 2 | 11 |
| B — Nurses | 48 | 1 | 4 | 6 | 2 | 4 | 7 | 72 |

(a) Burnout construct by group (included studies, k = 83)

→ The intern arm is the only arm containing academic-burnout studies; all registered-nurse studies measured occupational burnout. The crude between-group comparison therefore partly contrasts burnout construct with career stage. Restricting to occupational burnout in both arms removes this confound (see the Key comparison section).

(b) Burnout instrument family by group (included studies, k = 83)

→ Instrument distribution differs by group; because burnout instrument is itself a significant moderator (R² ≈ 21%, Moderators and subgroup analyses), career stage and instrument are statistically entangled, hence the instrument-adjusted meta-regression reported in the Moderators and subgroup analyses section.

**Supplementary Table S3. Characteristics and AHRQ methodological quality of all included studies (full version, with journal and region; k = 83).**

| **Study** | **Journal** | **Region** | **Group** | **N** | **Resilience** | **Burnout** | **r_total** | **r_EE** | **r_DP** | **r_PA** | **AHRQ** | **Quality** |
| --- | --- | --- | --- | --- | --- | --- | --- | --- | --- | --- | --- | --- |
| Gao (2025) (14) | Sci Rep | China | RN | 857 | CD-RISC | CMBI | -0.613 |  |  |  | 7 | Moderate |
| Chai (2025)(15) | Front Public Health | China | RN | 233 | CD-RISC | MBI-other | -0.661 | -0.743 | -0.672 | 0.677 | 8 | High |
| Yun (2022)(16) | Healthcare (Basel) | China | RN | 845 | work | MBI-other | -0.525 |  |  |  | 4 | Low |
| Gao (2023) (17) | Florence Nightingale J Nurs | China | RN | 717 | other | MBI-GS | -0.636 | -0.528 | -0.636 | 0.549 | 7 | Moderate |
| Huang (2025) (18) | BMC Nurs | China | RN | 1579 | CD-RISC | MBI-HSS |  | -0.361 | -0.237 | 0.556 | 6 | Moderate |
| Yan (2024) (19) | BMC Nurs | China | RN | 1627 | BRS | EE-only |  | -0.484 |  |  | 5 | Low |
| Lyu (2023) (20) | 昆明医科大学(硕士学位论文) | China | RN | 265 | CD-RISC | ProQOL | -0.203 |  |  |  | 7 | Moderate |
| Yang (2023)(21) | 赣南医学院(硕士学位论文) | China | RN | 300 | CD-RISC | MBI-GS |  | -0.132 | -0.158 | 0.486 | 8 | High |
| Guo JB (2019) (22) | 河南大学(硕士学位论文) | China | RN | 299 | CD-RISC | CMBI |  | -0.31 | -0.359 | 0.324 | 7 | Moderate |
| Sun YX (2019)(23) | 山东大学(硕士学位论文) | China | RN | 165 | CD-RISC | MBI-HSS |  | -0.054 | -0.212 | 0.626 | 8 | High |
| Sun YZ (2016)(24) | 安徽医科大学(硕士学位论文) | China | RN | 352 | CD-RISC | MBI-other |  | -0.741 | -0.698 | 0.703 | 8 | High |
| Ke (2024)(25) | 护理学杂志 | China | RN | 1574 | CD-RISC | MBI-GS | -0.571 |  |  |  | 7 | Moderate |
| Chen (2024)(26) | 军事护理 | China | RN | 527 | CD-RISC | EE-only |  | -0.582 |  |  | 7 | Moderate |
| Cui (2019) (27) | 护理研究 | China | RN | 730 | other | MBI-other | -0.49 | -0.34 | -0.39 | 0.37 | 5 | Low |
| Zou (2014) (28) | 护士进修杂志 | China | RN | 375 | CD-RISC | MBI-GS | -0.449 | -0.222 | -0.25 | 0.448 | 6 | Moderate |
| He (2009)(29) | 护理学杂志 | China | RN | 703 | hardiness | MBI-other |  | -0.182 | -0.397 | 0.351 | 6 | Moderate |
| Ou (2024)(30) | 苏州大学(硕士学位论文) | China | RN | 225 | CD-RISC | MBI-GS | -0.15 | -0.11 | -0.24 | 0.04 | 8 | High |
| Huang ZX (2019)(31) | 暨南大学(硕士学位论文) | China | RN | 394 | CD-RISC | MBI-GS | -0.567 | -0.287 | -0.402 | 0.549 | 7 | Moderate |
| Zhou (2020)(32) | 浙江大学(硕士学位论文) | China | RN | 203 | CD-RISC | MBI-GS | -0.47 | -0.14 | -0.12 | 0.66 | 7 | Moderate |
| Li (2015)(33) | 中华护理杂志 | China | RN | 192 | CD-RISC | MBI-GS | -0.495 | -0.405 | -0.39 | 0.379 | 5 | Low |
| Al-Harrasi (2024)(34) | J Perinat Neonatal Nurs | Other | RN | 173 | BRS | MBI-HSS |  | -0.26 | -0.176 | 0.218 | 7 | Moderate |
| Georges (2022)(35) | J Holist Nurs | Other | RN | 179 | CD-RISC | MBI-HSS |  | -0.17 | -0.007 | 0.263 | 5 | Low |
| Yildirim (2024)(36) | Int J Nurs Pract | Other | RN | 201 | BRS | other | -0.496 |  |  |  | 5 | Low |
| Guo (2018)(37) | J Clin Nurs | China | RN | 1061 | CD-RISC | MBI-GS |  | -0.271 | -0.311 | 0.317 | 6 | Moderate |
| Lang (2021)(38) | J Nurs Manag | Other | RN | 266 | CD-RISC | MBI-GS | -0.126 |  |  |  | 8 | High |
| Rushton (2015)(39) | Am J Crit Care | Other | RN | 114 | CD-RISC | MBI-HSS |  | -0.31 | -0.23 | 0.59 | 4 | Low |
| Luo (2022) (40) | J Nurs Manag | China | RN | 458 | RS | MBI-HSS | -0.51 | -0.43 | -0.33 | 0.39 | 9 | High |
| Yang (2018)(41) | Transplant Proc | China | RN | 536 | CD-RISC | MBI-other |  | -0.1 | -0.12 | 0.42 | 7 | Moderate |
| Lee (2025)(42) | J Nurs Manag | Other | RN | 605 | CD-RISC | CBI | -0.323 |  |  |  | 5 | Low |
| Rivas (2021)(43) | Int J Environ Res Public Health | Other | RN | 101 | CD-RISC | MBI-other |  | -0.271 |  | 0.288 | 4 | Low |
| Zhang (2024)‡(44) | J Korean Acad Nurs | China | Intern | 635 | CD-RISC | other | -0.57 |  |  |  | 7 | Moderate |
| Jiménez-Fernández (2022) (45) | J Nurs Manag | Other | RN | 375 | CD-RISC | MBI-other |  | -0.332 | -0.2 | 0.484 | 5 | Low |
| Abdulmohdi (2024)(46) | Nurs Open | Other | RN | 111 | CD-RISC | OLBI/OBI | -0.227 | -0.269 | -0.145 |  | 6 | Moderate |
| Ochs (2024)(47) | Nurs Educ Perspect | Other | RN | 25 | CD-RISC | MBI-student/educ |  | -0.631 | -0.619 | 0.491 | 3 | Low |
| Merino-Godoy (2022)(48) | Int J Ment Health Nurs | Other | Intern | 393 | CD-RISC | EE-only | -0.189 |  |  |  | 3 | Low |
| Abram (2021)(49) | Arch Psychiatr Nurs | Other | RN | 51 | RS | ProQOL | -0.39 |  |  |  | 6 | Moderate |
| Ríos-Risquez (2018)‡ (50) | J Adv Nurs | Other | Intern | 218 | CD-RISC | MBI-SS |  | -0.33 | -0.16 | 0.44 | 7 | Moderate |
| Zhang (2023)(51) | J Clin Nurs | China | RN | 319 | CD-RISC | MBI-other |  | -0.365 | -0.301 | 0.287 | 8 | High |
| Alkrenawi (2025)(52) | Nurse Educ | Other | Intern | 105 | CD-RISC | ProQOL | -0.27 |  |  |  | 7 | Moderate |
| Kolutek (2024)(53) | Arch Psychiatr Nurs | Other | RN | 945 | CD-RISC | CBI | -0.4 |  |  |  | 8 | High |
| LaPlante (2026)(54) | Dimens Crit Care Nurs | Other | RN | 218 | CD-RISC | other | -0.314 |  |  |  | 7 | Moderate |
| Zhang (2021)(55) | Int J Ment Health Nurs | China | RN | 180 | CD-RISC | MBI-GS | -0.387 | -0.283 | -0.4 | 0.388 | 5 | Low |
| Arrogante Maroto (2017) (56) | Rev Enferm | Other | RN | 133 | CD-RISC | MBI-HSS |  | -0.26 | -0.29 | 0.37 | 7 | Moderate |
| Guo (2019) (57) | J Clin Nurs | China | RN | 197 | CD-RISC | MBI-GS | -0.382 | -0.258 | -0.254 | 0.428 | 7 | Moderate |
| Alkubati (2025)(58) | Aust Crit Care | Other | RN | 306 | BRS | MBI-other |  | -0.48 | -0.34 | 0.16 | 8 | High |
| Min (2022) (59) | Medicine (Baltimore) | Other | RN | 155 | other | MBI-other | -0.58 |  |  |  | 5 | Low |
| Ogińska-Bulik (2021)(60) | Psychol Serv | Other | RN | 72 | other | OLBI/OBI | -0.59 | -0.5 |  |  | 7 | Moderate |
| García-Izquierdo (2017)(61) | J Nurs Scholarsh | Other | RN | 537 | CD-RISC | MBI-GS |  | -0.11 | -0.22 | 0.22 | 4 | Low |
| Jiménez-García (2026)(62) | Nurs Open | Other | RN | 256 | CD-RISC | MBI-HSS |  | -0.373 | -0.169 | 0.392 | 5 | Low |
| Gensimore (2020) (63) | J Nurs Adm | Other | RN | 501 | CD-RISC | MBI-HSS |  | -0.272 | -0.265 | 0.367 | 6 | Moderate |
| Ríos-Risquez (2016)‡ (64) | Contemp Nurse | Other | Intern | 113 | CD-RISC | MBI-SS |  | -0.51 | -0.2 | 0.35 | 7 | Moderate |
| Cha (2022) (65) | Int J Environ Res Public Health | Other | RN | 271 | CD-RISC | MBI-GS | -0.589 |  |  |  | 6 | Moderate |
| Jeong (2023)(66) | Intensive Crit Care Nurs | Other | RN | 147 | other | MBI-other | -0.54 |  |  |  | 6 | Moderate |
| Vieira (2022)(67) | Rev Lat Am Enfermagem | Other | RN | 153 | work | MBI-other |  | -0.545 | -0.419 | 0.68 | 5 | Low |
| Sikaras (2025)(68) | Behav Sci (Basel) | Other | RN | 380 | BRS | CBI | -0.457 |  |  |  | 6 | Moderate |
| Jose (2020)(69) | Indian J Crit Care Med | Other | RN | 120 | CD-RISC | MBI-HSS |  | -0.252 | -0.069 | 0.313 | 5 | Low |
| Zahednezhad (2021)(70) | Belitung Nurs J | Other | RN | 202 | CD-RISC | MBI-other | -0.5 | -0.46 | -0.26 | 0.37 | 6 | Moderate |
| Souza-Costa (2026)(71) | Nurs Res Pract | Other | RN | 239 | CD-RISC | MBI-other |  | -0.428 | -0.291 | 0.52 | 5 | Low |
| Rees (2016) (72) | Front Psychol | Other | Intern | 415 | CD-RISC | ProQOL | -0.486 |  |  |  | 7 | Moderate |
| Lee (2022)(73) | BMC Nurs | Other | RN | 146 | CD-RISC | MBI-other | -0.48 |  |  |  | 7 | Moderate |
| Jamebozorgi (2021) (74) | Front Psychiatry | Other | RN | 364 | CD-RISC | MBI-HSS |  | -0.442 |  | 0.351 | 5 | Low |
| Kutluturkan (2016)(75) | Ann Gen Psychiatry | Other | RN | 140 | RS | MBI-other |  | -0.191 | -0.161 | 0.35 | 6 | Moderate |
| Kchaou (2025)(76) | J Intensive Care Soc | Other | RN | 106 | BRS | ProQOL | -0.71 |  |  |  | 7 | Moderate |
| Baugh (2024)(77) | J Adv Pract Oncol | Other | RN | 27 | BRS | MBI-GS |  | -0.179 | -0.272 | 0.503 | 4 | Low |
| Hwang (2023) (78) | Front Psychol | Other | RN | 131 | CD-RISC | MBI-other | -0.51 |  |  |  | 8 | High |
| Shalom (2025)(79) | BMC Nurs | Other | RN | 465 | CD-RISC | CBI | -0.282 |  |  |  | 6 | Moderate |
| Miyazaki (2023)(80) | Healthcare (Basel) | Other | RN | 351 | other | other |  | -0.278 | -0.295 |  | 6 | Moderate |
| Altharman (2023) (81) | SAGE Open Nurs | Other | Intern | 564 | CD-RISC | MBI-other |  | -0.11 | -0.12 | 0.43 | 5 | Low |
| Suazo Galdames (2024)(82) | J Clin Med | Other | RN | 1013 | RS | MBI-HSS |  | -0.121 | -0.114 | 0.439 | 5 | Low |
| Lee (2019) (83) | Korean J Women Health Nurs | Other | Intern | 140 | other | MBI-GS | -0.36 |  |  |  | 6 | Moderate |
| Alia (2025)‡ (84) | Psychiatria | Other | Intern | 244 | other | other | -0.296 |  |  |  | 6 | Moderate |
| Buljubašić (2023)(85) | Psihološka otpornost i zadovoljstvo životom: Učinci sagorijevanja i posttraumatskog stresa u radnom okruženju pandemije COVID-19 | Other | RN | 125 | BRS | other | -0.258 |  |  |  | 5 | Low |
| Zanjani (2021) (86) | Iranian Journal of Psychiatry and Behavioral Sciences | Other | RN | 108 | CD-RISC | other | -0.38 |  |  |  | 5 | Low |
| Mohammadi (2021) (87) | Neuropsychiatria i Neuropsychologia | Other | RN | 420 | other | other |  | -0.7 |  |  | 7 | Moderate |
| Aghaei (2020) (88) | Iranian Red Crescent Medical Journal | Other | RN | 712 | other | MBI-other | -0.25 |  |  |  | 9 | High |
| Rísquez (2012)‡ (89) | Int J Psychol Res | Other | Intern | 218 | CD-RISC | MBI-GS |  | -0.248 | -0.063 | 0.521 | 6 | Moderate |
| Ghoneam (2025)(90) | TEACHING AND LEARNING IN NURSING | Other | Intern | 780 | CD-RISC | CBI | -0.097 |  |  |  | 7 | Moderate |
| Rubaca (2025) (91) | INTERNATIONAL JOURNAL OF ORGANIZATION THEORY & BEHAVIOR | Other | RN | 426 | BRS | EE-only |  | -0.618 |  |  | 7 | Moderate |
| Ata (2020) (92) | KONTAKT-JOURNAL OF NURSING AND SOCIAL SCIENCES RELATED TO HEALTH AND ILLNESS | Other | RN | 79 | RS | ProQOL | -0.424 |  |  |  | 6 | Moderate |
| Kim (2025)(93) | SAGE OPEN | Other | RN | 144 | CD-RISC | EE-only |  | -0.511 |  |  | 8 | High |
| Ertem (2020)(94) | CLINICAL AND EXPERIMENTAL HEALTH SCIENCES | Other | RN | 55 | CD-RISC | other | -0.542 | -0.707 | -0.351 | 0.567 | 4 | Low |
| Alonazi (2025) (95) | NURSING FORUM | Other | RN | 177 | CD-RISC | ProQOL | -0.21 |  |  |  | 5 | Low |
| Ko (2018)(96) | ASIAN ONCOLOGY NURSING | Other | RN | 157 | hardiness | ProQOL | -0.68 |  |  |  | 7 | Moderate |

*Note.* Same studies as main-text Table 1, with journal and region restored. ‡ academic-burnout study (nursing students), reported separately and not in the 78-study occupational analysis; blank = not reported. AHRQ = Agency for Healthcare Research and Quality 11-item score (High ≥8, Moderate 6–7, Low ≤5). Bracketed numbers are reference citations.

**Supplementary Table S4. Occupational-versus-academic burnout classification decision table for intern and student samples**

| **Study (year)** | **Role / sample** | **Burnout instrument** | **Construct measured** | **Assignment** |
| --- | --- | --- | --- | --- |
| Lee (2019) | Nursing intern (clinical placement) | MBI-GS | Occupational | Main occupational analysis (Group A) |
| Ghoneam (2025) | Nursing intern (clinical placement) | CBI | Occupational | Main occupational analysis (Group A) |
| Rees (2016) | Student nurse on placement | ProQOL | Occupational | Main occupational analysis (Group A) |
| Altharman (2023) | Nursing intern (clinical placement) | MBI (other) | Occupational | Main occupational analysis (Group A) |
| Merino-Godoy (2022) | Nursing intern (clinical placement) | Single-dimension EE-only | Occupational (EE-only) | Main occupational analysis (Group A); EE-only scale treated as the overall-burnout indicator |
| Alkrenawi (2025) | Nursing intern (clinical placement) | ProQOL | Occupational | Main occupational analysis (Group A) |
| Zhang (2024) | Nursing student (non-clinical) | Academic-burnout measure | Academic | Separately reported; excluded from main analysis |
| Ríos-Risquez (2018) | Nursing student (non-clinical) | MBI-Student Survey | Academic | Separately reported; excluded from main analysis |
| Ríos-Risquez (2016) | Nursing student (non-clinical) | MBI-Student Survey | Academic | Separately reported; excluded from main analysis |
| Alia (2025) | Nursing student (non-clinical) | Academic-burnout measure | Academic | Separately reported; excluded from main analysis |
| Rísquez (2012) | Nursing student (non-clinical) | MBI-Student Survey | Academic | Separately reported; excluded from main analysis |

Note. Classification rule: samples in a supervised clinical placement measured with an occupational-burnout instrument were assigned to the main occupational-burnout analysis (Group A); non-clinical nursing students measured with an academic/study-related burnout instrument (e.g., the MBI-Student Survey) were treated as out of scope and reported separately. A single-dimension emotional-exhaustion-only scale was treated as the study's overall-burnout indicator. EE, emotional exhaustion; MBI-GS, Maslach Burnout Inventory–General Survey; CBI, Copenhagen Burnout Inventory; ProQOL, Professional Quality of Life scale.

**Supplementary Figure Legend**

**Supplementary Fig S1.** Forest plot of the association between psychological resilience and total burnout (random-effects model; k = 44; pooled r = −0.44, 95% CI −0.48 to −0.39; I² = 92%).
